# Senescence-Associated Metabolomic Phenotype in Primary and iPSC-Derived Mesenchymal Stromal Cells

**DOI:** 10.1016/j.stemcr.2019.12.012

**Published:** 2020-01-23

**Authors:** Eduardo Fernandez-Rebollo, Julia Franzen, Roman Goetzke, Jonathan Hollmann, Alina Ostrowska, Matteo Oliverio, Torsten Sieben, Björn Rath, Jan-Wilhelm Kornfeld, Wolfgang Wagner

**Affiliations:** 1 Helmholtz-Institute for Biomedical Engineering, Stem Cell Biology and Cellular Engineering, RWTH Aachen University Medical School, Aachen 52074, Germany; 2 Institute for Biomedical Technology – Cell Biology, RWTH Aachen University Medical School, Aachen 52074, Germany; 3 Max Planck Institute for Metabolism Research (MPI-MR), Noncoding RNAs and Energy Homeostasis, Gleueler Strasse 50, Cologne 50931, Germany; 4 Cologne Cluster of Excellence: Cellular Stress Responses in Aging-Associated Diseases (CECAD), Cologne 50931, Germany; 5 Department for Orthopedics, RWTH Aachen University Medical School, Aachen 52074, Germany; 6 University of Southern Denmark, Functional Genomics and Metabolism Unit, Department for Biochemistry and Molecular Biology, Campusvej 55, Odense 5230, Denmark

**Keywords:** replicative senescence, metabolomics, mesenchymal stromal cells, induced pluripotent stem cells, transcriptomics, DNA methylation

## Abstract

Long-term culture of primary cells is characterized by functional and secretory changes, which ultimately result in replicative senescence. It is largely unclear how the metabolome of cells changes during replicative senescence and if such changes are consistent across different cell types. We have directly compared culture expansion of primary mesenchymal stromal cells (MSCs) and induced pluripotent stem cell-derived MSCs (iMSCs) until they reached growth arrest. Both cell types acquired similar changes in morphology, *in vitro* differentiation potential, senescence-associated β-galactosidase, and DNA methylation. Furthermore, MSCs and iMSCs revealed overlapping gene expression changes, particularly in functional categories related to metabolic processes. We subsequently compared the metabolomes of MSCs and iMSCs and observed overlapping senescence-associated changes in both cell types, including downregulation of nicotinamide ribonucleotide and upregulation of orotic acid. Taken together, replicative senescence is associated with a highly reproducible senescence-associated metabolomics phenotype, which may be used to monitor the state of cellular aging.

## Highlights

• MSCs and iPSC-derived MSCs (iMSCs) acquired functional changes upon senescence
• Senescence of MSCs and iMSCs evoked similar transcriptomic changes
• Senescence of MSCs and iMSCs was associated with similar metabolomic phenotypes

## Graphical Abstract

**Figure:**
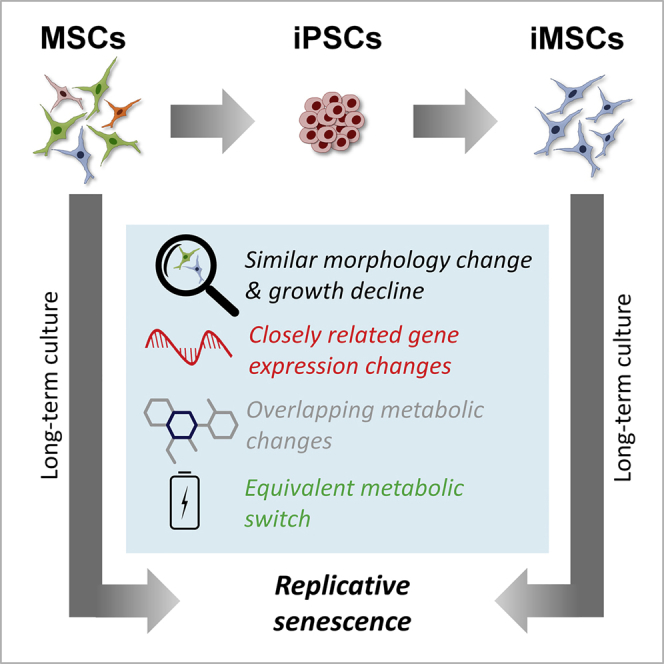

## Teaser

Wagner and colleagues show that expansion of iPSC-derived MSCs evokes molecular, functional, and metabolic changes similar to those observed in their primary counterparts. The highly consistent changes indicate that the metabolic phenotype can be used as a biomarker to assess the state of cellular aging, and this is relevant for regenerative medicine.

## Introduction

*In vitro* culture of primary cells is associated with continuous changes that ultimately result in replicative senescence: the proliferation rate declines, cells enlarge, and they lose differentiation potential (Campisi and d’Adda di Fagagna, 2007). These profound changes in the course of culture expansion hamper the reproducibility of experiments, which is of particular relevance in regenerative medicine (Wagner and Ho, 2007). For example, mesenchymal stromal cells (MSCs) acquire continuous changes in gene expression and DNA methylation over subsequent passages, which can be used to track the state of cellular aging (Koch et al., 2012, Li et al., 2017, Schellenberg et al., 2014, Wagner et al., 2008). In contrast to quiescent or apoptotic cells, senescent cells are highly metabolically active (Wiley and Campisi, 2016). Senescent cells secrete a characteristic cocktail of interleukins, chemokines, and growth and inflammatory factors, which compose the senescence-associated secretory phenotype (SASP) (Coppé et al., 2008). The SASP was meanwhile observed across multiple cell types, and there is evidence that this secretory function of senescent cells has an impact on wound healing, embryonic development, and tumorigenesis (Watanabe et al., 2017). However, little is known about the metabolomic changes that may be associated with or even contribute to the process of cellular aging.

In contrast to primary cells, such as MSCs, induced pluripotent stem cells (iPSCs) do not reveal any signs of replicative senescence (Koch et al., 2013, Lapasset et al., 2011). They can be culture expanded for many passages without reduced proliferation, loss of differentiation potential, or telomere attrition. It is unclear how iPSCs escape from replicative senescence, while there is evidence that their progeny are bound to this destiny again upon exit from the pluripotent state (Frobel et al., 2014). Long-term growth curves and the molecular sequel of replicative senescence have so far hardly been systematically addressed in iPSC-derived cells. A better understanding of cellular aging upon differentiation of iPSCs is therefore urgently needed with regard to the enormous hopes for regenerative medicine.

Various protocols have been described for generating iPSC-derived MSC-like cells (iMSCs) (Diederichs and Tuan, 2014, Frobel et al., 2014, Kang et al., 2015). These cells closely resemble their primary counterparts in morphology, immunophenotype, and three-lineage differentiation potential toward osteocytes, chondrocytes, and adipocytes (Sabapathy and Kumar, 2016). It has been suggested that iMSCs might be more homogeneous than primary MSCs, which are well known to comprise multiple subpopulations (Ho et al., 2008). However, on the epigenetic level MSCs and iMSCs still remained distinct and DNA methylation patterns that reflect the tissue of origin or donor age were not recapitulated in iMSCs (Frobel et al., 2014). So far, it was unclear whether iMSCs undergo the same molecular changes as primary MSCs during culture expansion. In this study, we have therefore compared functional, transcriptomic, and metabolomic changes during long-term growth of MSCs and iMSCs.

## Results

### iPSC-Derived Mesenchymal Stromal Cells Are Bound to Replicative Senescence

**Figure 1:**
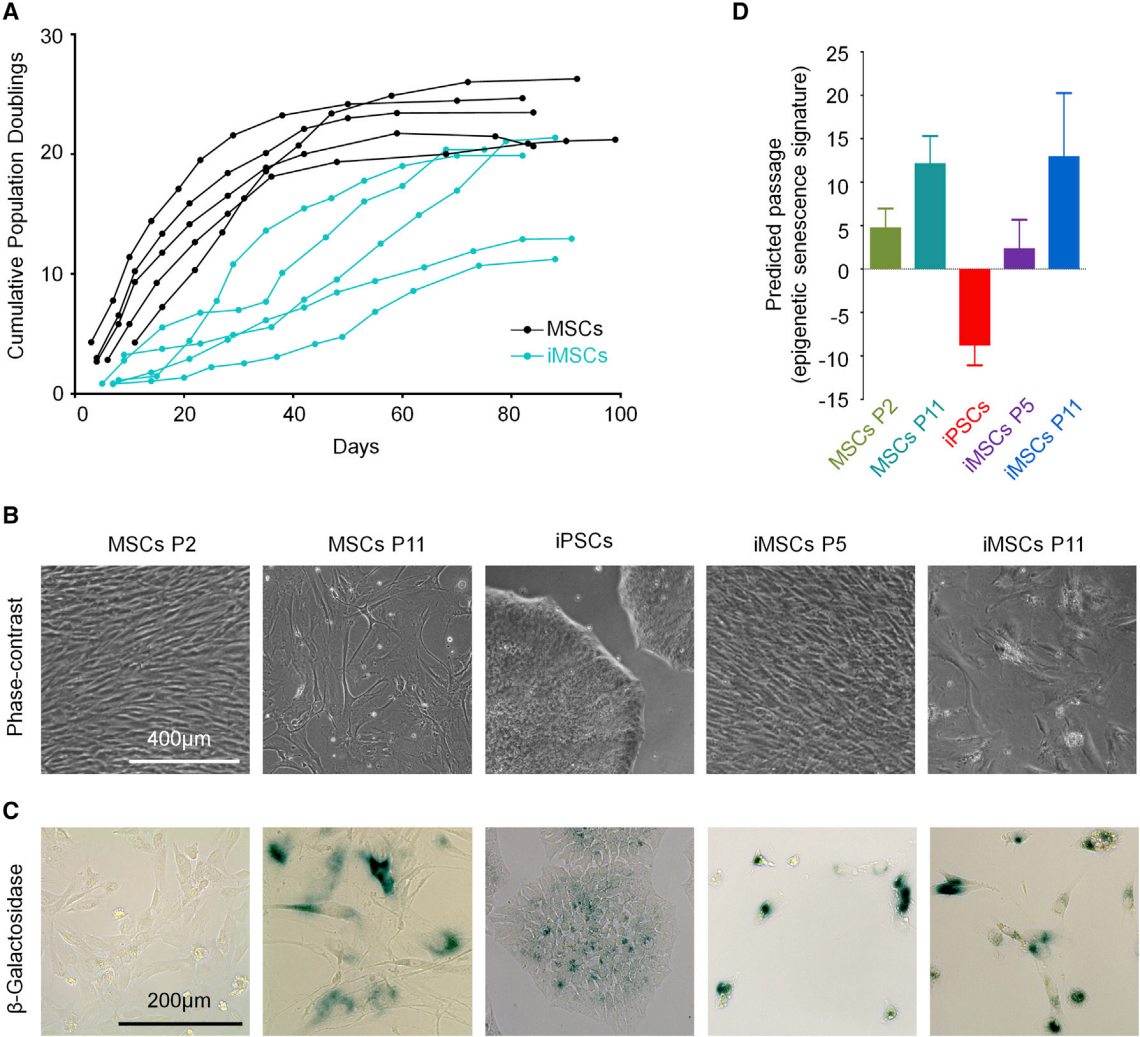
Replicative Senescence of iPSC-derived MSCs (A) Long-term growth curves of syngeneic MSCs and iMSCs demonstrate that both cell types enter proliferative arrest within 3 months. (B) Phase-contrast images of MSCs (passages 2 and 11) and iPSCs and iMSCs (passages 5 and 11). (C) Staining for senescence-associated β-galactosidase was more pronounced at later passages, albeit several iPSCs and iMSCs at early passage were also detected positive. (D) The number of passages was estimated based on an epigenetic senescence signature that utilizes DNA methylation levels at six CG dinucleotides (n = 5 biological replicates) (Koch et al., 2012).

MSCs at first passage (n = 5) were reprogrammed into iPSCs and then redifferentiated toward MSCs (iMSCs). Syngeneic MSCs and iMSCs were subsequently expanded until the cells entered proliferation arrest. MSC proliferation rates decreased after about 20–40 days, whereas iMSCs proliferated more slowly, with a later decline in proliferation rate. Within 3 months all cell populations reached the senescent state, with a mean number of population doublings of 21.3 ± 1.4 and 17.1 ± 3.8 for MSCs and iMSCs, respectively (Figure 1A). The changes in cellular morphology were very comparable between MSCs and iMSCs: at early passages they displayed spindle-shaped fibroblast-like morphology, whereas cells at later passages were enlarged, with flattened “fried egg” morphology (Figure 1B).

The immunophenotypes of MSCs and iMSCs were similar (CD29^+^, CD73^+^, CD90^+^ CD105^+^, CD14^−^, CD31^−^, CD34^−^, and CD45^−^) and remained stable throughout culturing (Figure S1A). MSCs and iMSCs of early passages could be differentiated *in vitro* toward osteogenic, adipogenic, and chondrogenic lineages (Frobel et al., 2014). The differentiation potential of MSCs decayed at later passages, as described before (Wagner et al., 2008), and this was also observed for iMSCs (Figure S1B).

Both cell types revealed increased staining for senescence-associated β-galactosidase (SA-β-gal) at later passages, albeit several iPSCs and iMSCs at early passages also stained positive for SA-β-gal (Figure 1C). Subsequently, we estimated the state of cellular aging based on epigenetic modifications. We previously demonstrated that the number of passages is reflected by concurring DNA methylation changes at six senescence-associated CG dinucleotides (Franzen et al., 2017, Koch et al., 2012). MSCs and iMSCs revealed increasing epigenetic senescence predictions at later passages, whereas iPSCs were even predicted to be of negative passages (Figure 1D). Taken together, iMSCs fulfilled the minimal criteria for definition of MSCs (Dominici et al., 2006), and both cell types apparently reveal very similar modifications during long-term culture that ultimately result in replicative senescence.

### Senescence-Associated Gene Expression Is Closely Related in MSCs and iMSCs

**Figure 2:**
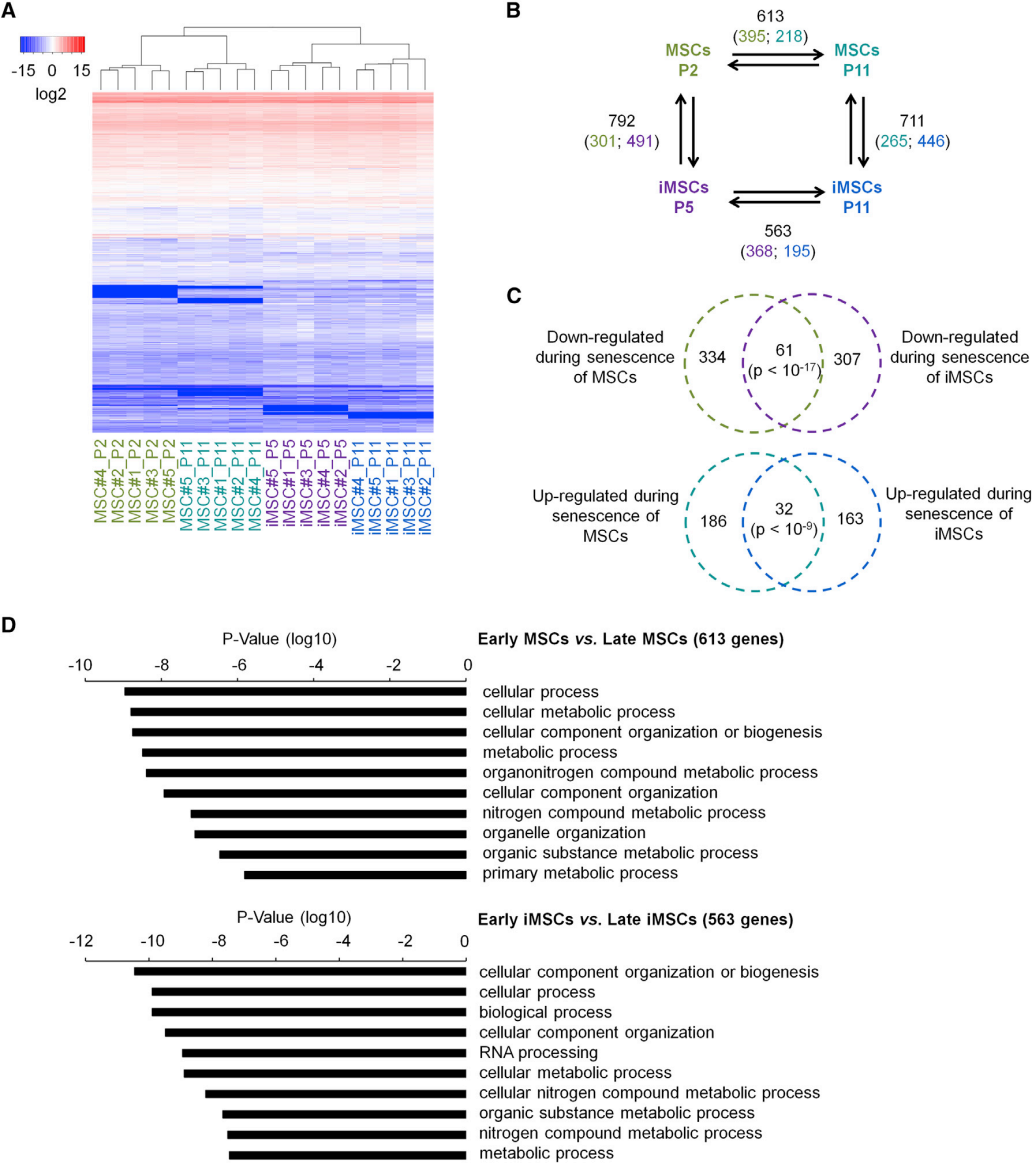
Transcriptome Analysis of Replicative Senescence (A) Heatmap of the entire transcriptome of MSCs and corresponding iMSCs (n = 5, donor numbers are indicated). Hierarchical clustering clearly separated early and late passages for both cell types. (B) The numbers of transcripts with significant gene expression changes in pairwise comparisons are depicted (2-fold differential expression and limma adjusted p < 0.05; the number of upregulated genes in the corresponding cell type is indicated by color code). (C) The Venn diagrams depict significant overlaps of down- and upregulated transcripts during senescence of MSCs and iMSCs (significance was estimated by hypergeometric distribution). (D) The senescence-associated gene expression changes in MSCs and iMSCs were classified by Gene Ontology analysis. Functional categories involved in metabolic processes were among the most significantly overrepresented (Fisher t test).

It has been demonstrated that long-term culture of MSCs is reflected by unique changes in their gene expression profiles (Wagner et al., 2008). To address the question of whether iMSCs reflect similar modifications we compared the transcriptomes of MSCs and iMSCs at early and late passages by deep sequencing (n = 5). Overall the gene expression profiles of MSCs and iMSCs were closely related, albeit they were clearly separated by hierarchical clustering regarding cell types and passages (Figure 2A).

Pairwise comparison of MSCs and iMSCs revealed significant differences between early and late passages (2-fold differential expression and adjusted p < 0.05): 613 and 563 transcripts were differentially expressed upon senescence of MSCs and iMSCs, respectively (Figure 2B; Table S1). We did not observe consistent upregulation of SASP-associated genes (Figure S1C). Notably, senescence-associated changes in MSCs and iMSCs share a relatively high number of downregulated transcripts (61 transcripts; hypergeometric distribution: p < 10^−17^) and upregulated transcripts (32 transcripts; p < 10^−9^; Figure 2C). Senescence-associated gene expression changes were particularly enriched in Gene Ontology (GO) categories related to metabolism, such as cellular metabolic process, nitrogen compound metabolic process, or organic substance metabolic process, among others (Figure 2D). Genes associated with the GO category metabolic process (GO: 0008152) are highlighted in Table S1. These results indicate that metabolic changes might be of particular relevance for the process of cellular aging in MSCs and iMSCs.

### Senescence Is Reflected by Consistent Changes in the Metabolome

**Figure 3:**
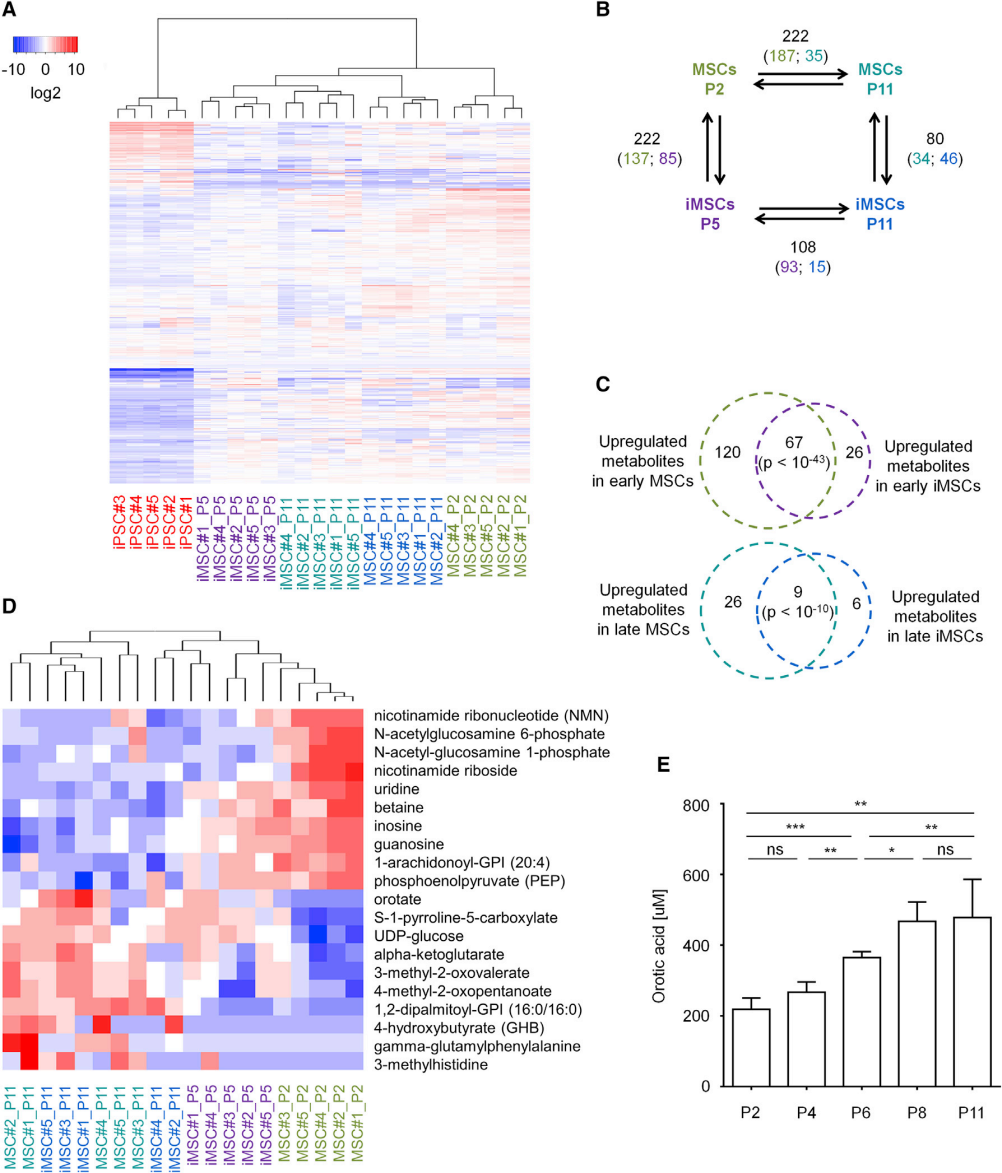
Metabolomic Characterization of Replicative Senescence (A) Heatmap presentation of metabolomes (612 different metabolites). Hierarchical clustering clearly separated iPSCs, MSCs, and iMSCs, as well as cells at early and late passage (n = 5, donor numbers are indicated). (B) Schematic presentation of the numbers of metabolites with significant differences in pairwise comparisons (1.5-fold difference and adjusted p < 0.05; higher abundance is indicated by the color code of corresponding cell type). (C) The Venn diagrams depict a high overlap of senescence-associated changes in MSCs and iMSCs for upregulated and downregulated metabolites (significance was estimated by hypergeometric distribution). (D) Heatmap of the ten most significant up- and downregulated metabolites during replicative senescence (MSCs + iMSCs). (E) Orotic acid was exemplarily measured by a fluorogenic assay in MSCs (n = 4 biological replicates) and increased over subsequent passages, which is in line with our metabolomics results. * p. < 0.05; ** p < 0.005 and *** p < 0.001.

To gain insight into how the metabolism varies during long-term culture, we analyzed the metabolomes of MSCs at passage 2 (n = 5) and passage 11 (n = 5) and of iMSCs at passage 5 (n = 5) and passage 11 (n = 5). For comparison we also analyzed the five corresponding iPSC preparations. Using chromatographic approaches (ultra-high-performance liquid chromatography/mass spectrometry/mass spectrometry [UHPLC/MS/MS] and gas chromatography/mass spectrometry [GC/MS]), 612 different metabolites were identified and measured (Table S2). Hierarchical clustering of metabolomic profiles clearly separated cell types as well as early and late passages. In fact, MSCs and iMSCs at passage 11 comprised similar metabolites (Figure 3A).

We subsequently analyzed if there were significant differences in the metabolomic profiles of cells at early and late passages (>1.5-fold difference and adjusted p < 0.05): 222 and 108 metabolites were significantly changed in MSCs and iMSCs, respectively (Figure 3B; Table S2). Notably, there was a highly significant overlap of senescence-associated metabolic changes in MSCs and iMSCs: 67 metabolites were consistently downregulated (hypergeometric distribution: p < 10^−43^) and 9 metabolites were upregulated at later passages in both cell types (p < 10^−10^; Figure 3C). In contrast, only sphingomyelin was significantly upregulated during senescence of MSCs and downregulated in iMSCs, while inosine and uridine metabolites were upregulated during senescence of iMSCs and downregulated in MSCs. These results demonstrate that long-term culture of MSCs and iMSCs is overall associated with similar metabolomic changes. Among the metabolites with the most drastic downregulation in senescence were N-acetylglucosamine 6-phosphate, N-acetylglucosamine 1-phosphate, nicotinamide riboside, and nicotinamide ribonucleotide, whereas upregulated metabolites included orotate, gamma-glutamylphenylalanine, 4-hydroxybutyrate, and 3-methylhistidine (Figure 3D). Taken together, replicative senescence is associated with significant metabolomic changes that are similar in MSCs and iMSCs.

We have exemplarily validated the senescence-associated changes in orotic acid in cell pellets of MSCs (n = 4) using a fluorogenic reaction (Yin et al., 2015). The results validated a significant increase in orotic acid over subsequent passages (Figure 3E). Orotic acid is needed for pyrimidine synthesis and, thus, the increase might result from DNA/RNA synthesis abrogation. However, the RNA concentration rather increased in later passages, and senescent cells were often stalled in G2 (Figures S2A and S2B). To further analyze if increasing concentrations of orotic acid might be functionally relevant for senescence, we performed additional long-term culture experiments with culture medium that was supplemented with 1 mM orotic acid, but there was no consistent acceleration of replicative senescence (Figures S2C and S2D). While these results indicate that orotic acid is not functionally relevant for the process of cellular senescence, the quantification of specific metabolites can be used to track cellular aging.

### The Bioenergetic Switch in Senescence

**Figure 4:**
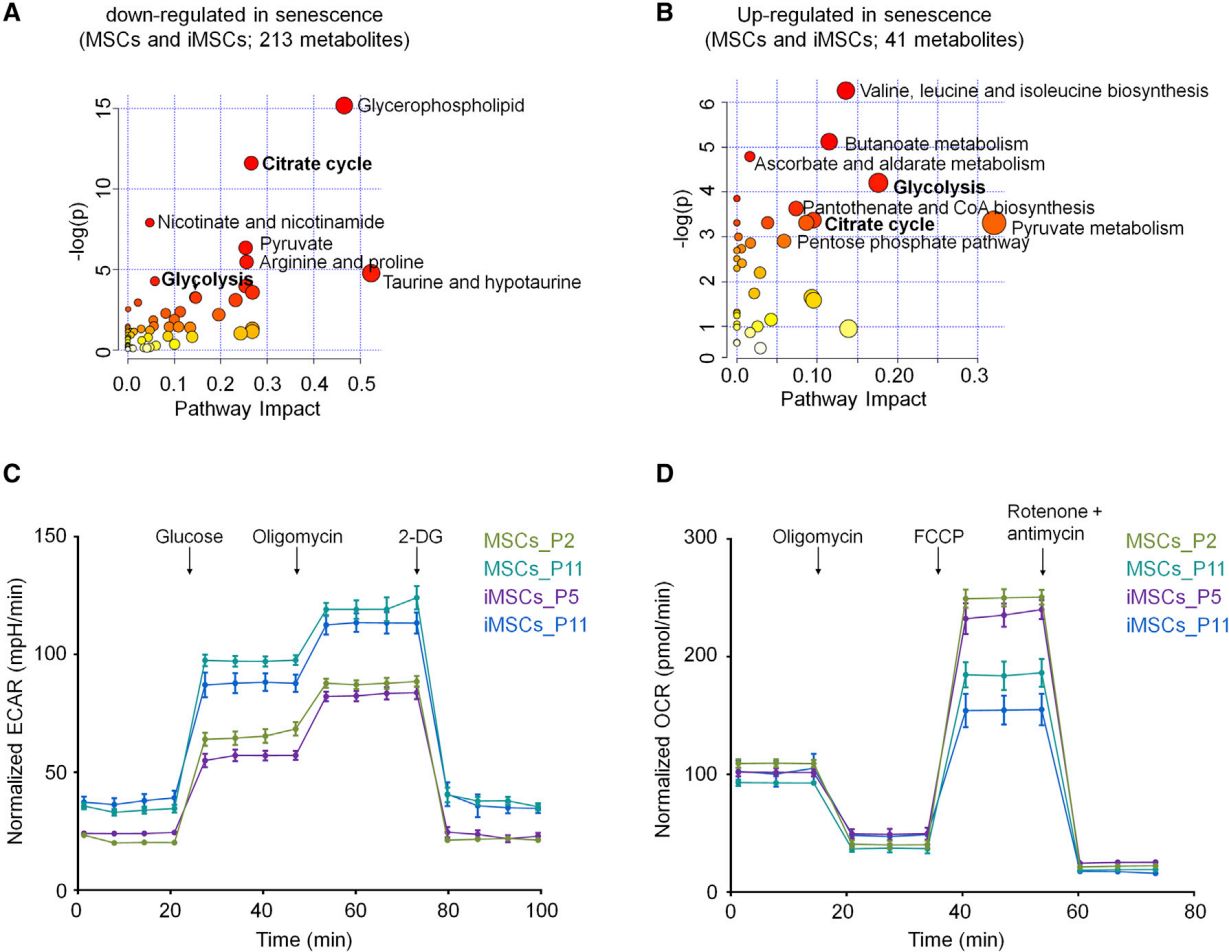
Metabolic Pathway Analysis during Replicative Senescence (A and B) (A) The metabolic pathway analysis (using MetaboAnalyst 4.0) indicated that the 213 metabolites that were significantly downregulated during senescence of MSCs and/or iMSCs pointed toward more oxidative metabolism, whereas (B) the 41 upregulated metabolites were rather related to glycolytic metabolism. Scores for enrichment (vertical axis) and topology analyses (pathway impact, horizontal axis) are depicted (color code depicts overall significance, and the size of the circles reflects centrality of the involved metabolites). (C and D) (C) Metabolic flux analysis (Seahorse Bioscience) of the extracellular acidification rate (ECAR) indicates that senescent MSCs and iMSCs use more glycolysis, while (D) the oxygen consumption (OCR) indicates higher oxidative metabolism in MSCs and iMSCs of early passages (n = 3 biological replicates and n = 8 technical replicas for each condition).

To gain better insight into how the senescence-associated metabolites map to specific metabolic pathways, we used MetaboAnalyst (Xia et al., 2015). The identified pathways were similar for MSCs and iMSCs and even had a similar enrichment for up- and downregulated metabolites, which may indicate that the relevant proteins of these pathways are regulated independently (Figure S3). For simplicity, we subsequently focused on all metabolites that were at least significantly changed in MSCs and/or iMSCs: 213 downregulated metabolites were particularly associated with the glycerophospholipid pathway (p = 2.6 × 10^−7^ and pathway impact score [PI] = 0.47) and the tricarboxylic acid cycle (p = 9.2 × 10^−6^ and PI = 0.27), as well as the taurine and hypotaurine pathways (p = 0.0084 and PI = 0.52; Figure 4A). On the other hand, the 41 upregulated metabolites were enriched in the biosynthesis of valine, leucine, and isoleucine (p = 0.0019 and PI = 0.14), butanoate metabolism (p = 0.0060 and PI = 0.11), pyruvate metabolism (p = 0.0083 and PI = 0.017), and glycolysis (p = 0.015 and PI = 0.18; Figure 4B). These results indicated that changes in the tricarboxylic acid cycle and glycolysis might be of particular relevance during senescence. Overall, these pathways seemed to be hardly regulated on the gene expression level (Figures S4A and S4B), but on the protein level, we observed a significant upregulation of hexokinase II at late passages of MSCs and iMSCs, which provides additional evidence for the enhanced glycolytic state (Figure S4C).

In fact, previous studies indicated that various cell types gradually shift toward a more glycolytic state during culture expansion (Bittles and Harper, 1984, Zwerschke et al., 2003). We therefore analyzed if this metabolic switch occurs also in MSCs and iMSCs using a flux analyzer to monitor the extracellular acidification rate (ECAR) and the mitochondrial oxygen consumption rate (OCR). ECAR rather reflects glycolytic behavior and it was consistently higher in senescent MSCs and iMSCs (Figure 4C). In contrast, OCR was higher and more affected in early passages of MSCs and iMSCs (Figure 4D). Since mitochondrial function might be impaired, we also analyzed the amount of reactive oxygen species (ROS), which are by-products of the respiratory chain. We observed only a very moderate upregulation of ROS in later passages (Figure S4D). Taken together, MSCs and iMSCs reveal the same senescence-associated shift to glycolytic pathways upon senescence.

## Discussion

Recently, the first clinical trials with iMSCs were started for steroid-refractory graft versus host disease (Rasko et al., 2019), and it will be important to understand if these cell preparations are equally as safe and potent as primary MSCs. In this context, it is also crucial to better understand how culture expansion affects iMSCs. The results of this study indicate that upon exit from the pluripotent state the iMSCs undergo the same functional and molecular changes as previously described for primary MSCs: iMSCs recapitulate senescence-associated changes in morphology, loss of differentiation potential, and ultimately growth arrest. Furthermore, the senescence markers SA-β-gal and the epigenetic senescence signature increased with serial passaging. It might have been anticipated that iMSCs, which are derived from entirely rejuvenated iPSCs, could reach a higher number of cumulative population doublings, whereas our results indicate that iMSCs enter the senescent state after fewer cumulative population doublings even than primary MSCs. On the other hand, the state of cellular aging may be better controlled in iPSC-derived cells by the possibility of expanding iPSCs in the pluripotent state to then generate iMSCs of low passage in relevant cell numbers.

Only a few studies have investigated metabolomic changes during culture expansion, and most of them addressed changes in fibroblasts (James et al., 2015, Zwerschke et al., 2003). Overall, there was a consistent shift from oxidative phosphorylation toward glycolysis and the pentose phosphate pathway (James et al., 2016, Zwerschke et al., 2003). The metabolic switch might be relevant to counteract extensive accumulation of ROS levels. The increase in these reactive intermediates might be attributed to the finding that mitochondria at later passages are often dysfunctional (Zhang et al., 2018). An alternative explanation for the metabolic shift might be a vicious cycle, where depletion of ATP leads to an adaptive response that increases metabolic imbalance (Zwerschke et al., 2003).

Our results demonstrate that replicative senescence has a major impact on the metabolome, with highly reproducible changes across different cell types and biological replica. Lee et al. have previously identified eight metabolites with altered expression during senescence of MSCs, including lysophosphatidylcholine and lysophosphatidylethanolamine (Lee et al., 2014), while we describe a multitude of significant changes. At this point, it is unclear if the senescence-associated metabolic changes are functionally relevant *per se* or rather a consequence of other modifications, e.g., on chromatin or the transcriptome level. At least for orotic acid, we did not observe a clear impact on the senescence process. Either way, the highly consistent changes indicate that the metabolic phenotype can be used as a biomarker to assess the state of cellular aging.

## Experimental Procedures

### Cell Culture of Mesenchymal Stromal Cells

MSCs were isolated from the bone marrow of five donors (ranging from 50 to 74 years of age) after orthopedic hip replacement surgery. All samples were taken after written consent according to the guidelines of the local ethics committees (RWTH Aachen; EK300/13) and isolated as described before (Koch et al., 2013). In brief, cells were flushed from the bone and cultured in parallel in basal medium consisting of Dulbecco's modified Eagle’s medium (1 g/L glucose; PAA, Pasching, Austria), with 1% penicillin/streptomycin (PAA) and 1% L-glutamine, and supplemented with 10% human platelet lysate (HPL). HPL pools consisted of at least five lysates to reduce variation, and coagulation was prevented by 0.61 IU unfractionated heparin (Ratiopharm, Ulm, Germany).

### Generation of iPSCs and Differentiation toward MSCs

IPSCs were reprogrammed from the same MSC donors that were used for experiments with primary MSCs. Reprogramming was performed with episomal plasmids (Willmann et al., 2013). iPSCs were cultured on tissue culture plastic coated with vitronectin (0.5 μg/cm^2^) in StemMACS iPS-Brew XFe96 (all Miltenyi Biotec, Bergisch Gladbach, Germany). Pluripotency was validated by *in vitro* differentiation and Epi-Pluri-Score (Cygenia, Aachen, Germany) (Lenz et al., 2015). iPSCs were redifferentiated toward iMSCs under MSC culture conditions as described above (Frobel et al., 2014). After 1 week, differentiated iPSCs were maintained on 0.1% gelatin-coated TCP or HPL-gel and passaged every further week using trypsin-EDTA 0.25% (Gibco/Thermo Fisher Scientific).

### Metabolomics

Pellets of 3 × 10^6^ cells were subjected to methanol extraction and then split into aliquots for analysis by UHPLC/MS/MS and GC/MS (Tufi et al., 2014). Metabolomics analysis was conducted at Metabolon (Durham, NC, USA) as previously described (Long et al., 2017). Briefly, metabolites were identified by automated comparison of ion features to a reference library of chemical standards followed by visual inspection for quality control. Missing values were assumed to be below the detection limits and imputed with the compound minimum (minimum value imputation). To identify the most relevant metabolic pathways during replicative senescence we employed the pathway enrichment analysis for the up- and downregulated metabolites in MSCs and iMSCs using the pathway analysis tool from MetaboAnalyst 4.0 (www.metaboanalyst.ca). The pathway enrichment analysis used GlobalTest to analyze the concentration values with high sensitivity and to identify subtle changes involved in the same biological pathway (Chong et al., 2018). Statistical tests were performed using the R Bioconductor package and significance was estimated using one-way ANOVA and adjusted p < 0.05.

Additional information on flow cytometry, proliferation analysis, *in vitro* differentiation of MSCs and iMSCs, transcriptomics, quantification of orotic acid, western blot, metabolic flux assay, and ROS detection is provided in the Supplemental Information.

## Author Contributions

E.F.-R. and W.W. developed the study concept and experimental design. E.F.-R., J.F., R.G., J.H., A.O., T.S., and M.O. performed the experiments and interpreted the data. J.-W.K. contributed vital reagents and materials and provided important intellectual support throughout the study. E.F.-R. and W.W. wrote the first draft of the manuscript. All authors read and approved the final manuscript.

## Conflicts of Interest

W.W. is cofounder of Cygenia GmbH (www.cygenia.com), which may provide service for the epigenetic senescence signature to other scientists. R.G. and J.F. also contribute to this company.

## Supplemental Information

Document S1. Supplemental Experimental Procedures and Figures S1–S4

Table S1. Differentially Expressed Transcripts upon Senescence of MSCs and iMSCs, Related to Figure 2

Table S2. Identified Metabolites, Related to Figure 3

Document S2. Article plus Supplemental Information
