# Supplementary material for: Senescence-Associated Metabolomic Phenotype in Primary and iPSC-Derived Mesenchymal Stromal Cells
Source: Stem Cell Reports. 2020 Jan 23;14(2):201–9. doi: 10.1016/j.stemcr.2019.12.012 (PMC7013233; doi:10.1016/j.stemcr.2019.12.012)
Supplement: Document S1. Supplemental Experimental Procedures and Figures S1–S4 [file mmc1.pdf]

Stem Cell Reports, Volume *14*

Supplemental Information

Senescence-Associated Metabolomic Phenotype in Primary and iPSC-Derived Mesenchymal Stromal Cells

Eduardo Fernandez-Rebollo, Julia Franzen, Roman Goetzke, Jonathan Hollmann, Alina Ostrowska, Matteo Oliverio, Torsten Sieben, Björn Rath, Jan-Wilhelm Kornfeld, and Wolfgang Wagner

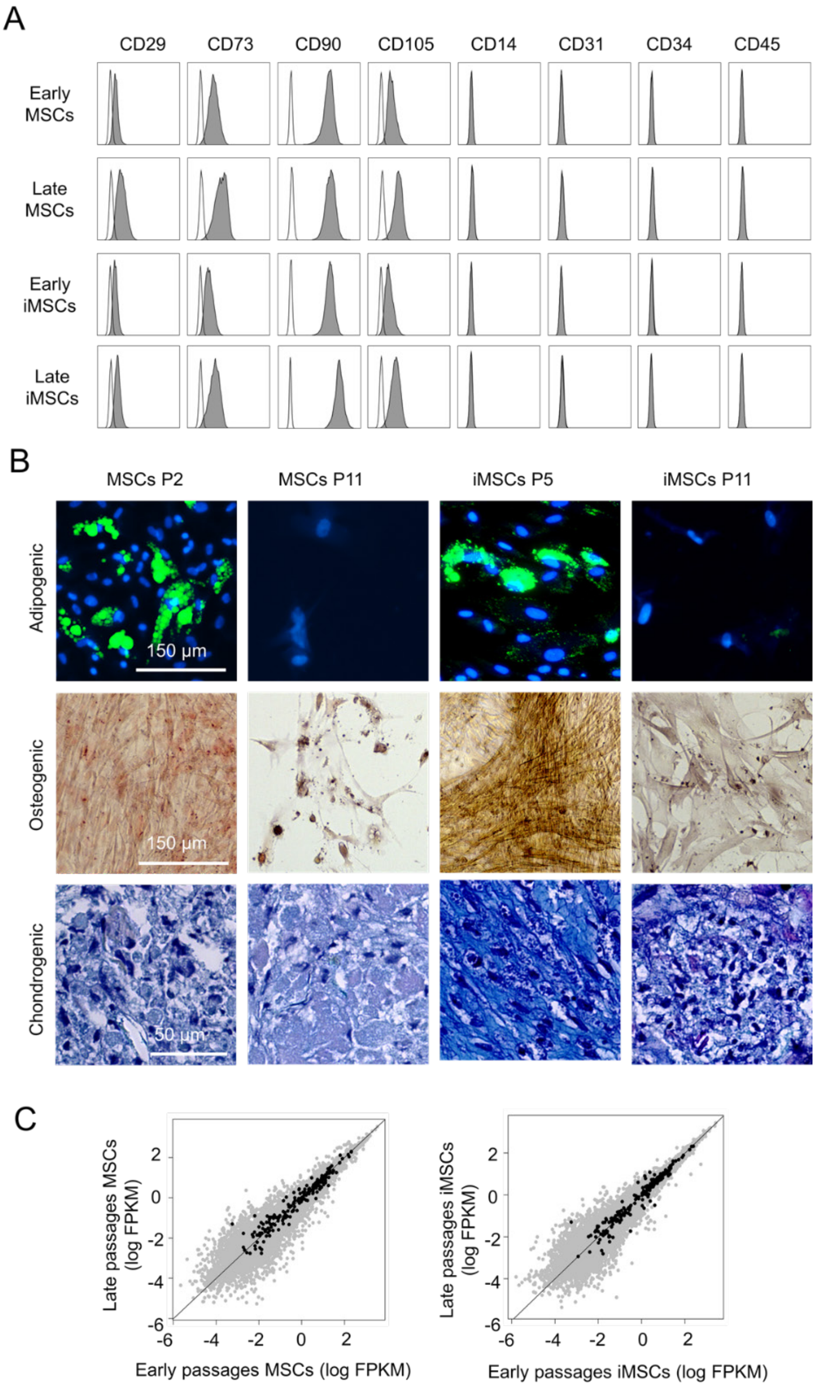

Figure S1: Immunophenotype and Differentiation Potential of MSCs and iMSCs, Related to Figure 1. **(A)** Histograms depict exemplarily the immunophenotype of MSCs and iMSCs in early and late passages. **(B)** MSCs and iMSCs in early and late passages were differentiated towards adipogenic, osteogenic and chondrogenic lineages and analyzed as indicated in the text. **(C)** Expression of the genes involved in the senescence associated secretory phenotype (SASP). To that end we curated a list of genes - combining the list of genes from http://www.saspatlas.com/, https://reactome.org and https://www.labome.com/, which have been associated to SASP. Scatter plot of early versus late passages in MSCs (left) and iMSCs (right) indicated that SASP associated genes (black dots) are not generally upregulated as compared to other genes (grey). There was overall little up-regulation of genes for SASP proteins, which might be attributed to difference between gene expression and protein level, and to heterogeneity amongst cell types and time points (Hernandez-Segura et al., 2017).

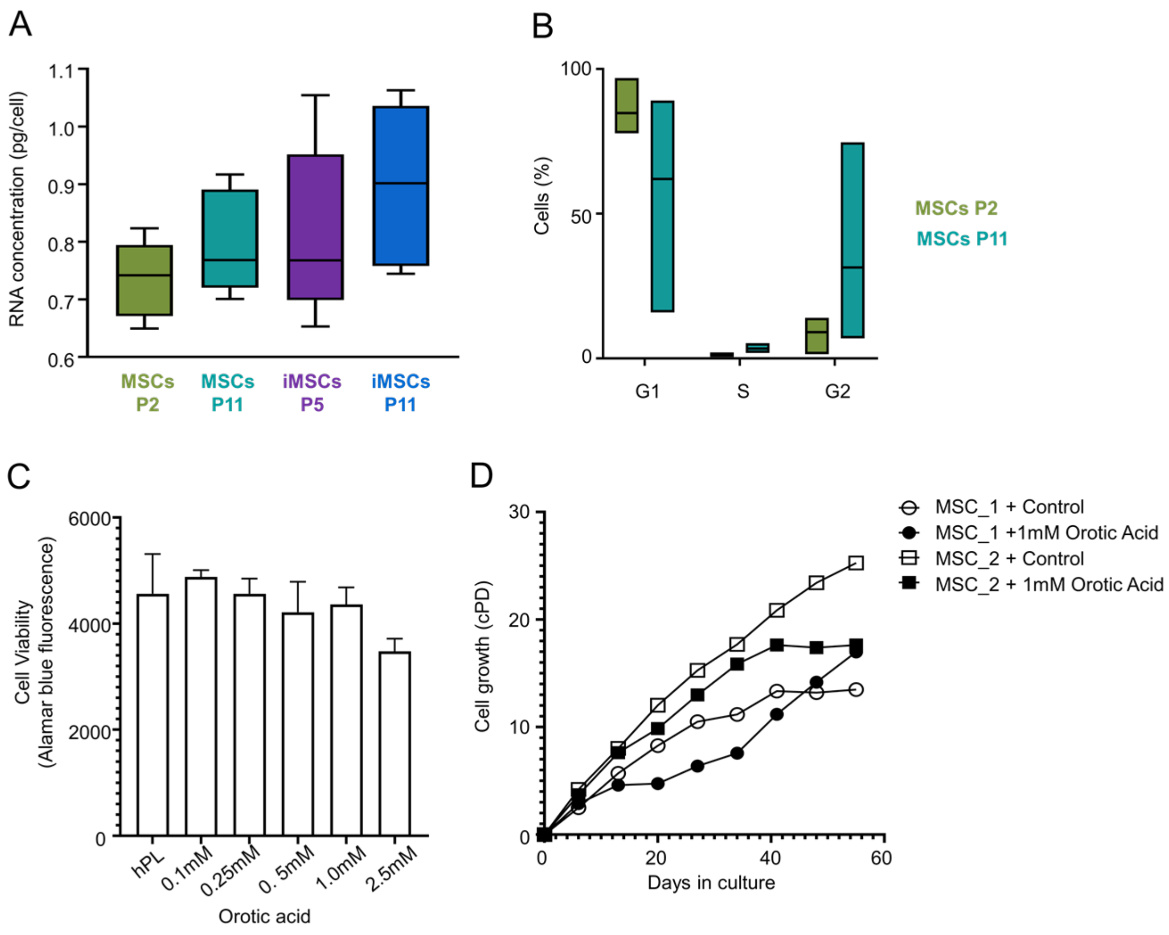

Figure S2: Cell Cycle Arrest and Culture with Orotic Acid, Related to Figure 3.
It has been suggested that nucleotide synthesis inhibition plays a causative role in the establishment of replicative senescence in human mammary epithelial cells (Delfarah et al., 2019). Therefore, we analyzed the RNA concentration per cell, the cell cycle, and the impact of orotic acid, which is needed for pyrimidine synthesis. **(A)** Box plot representation for normalized RNA content (pg/cell) for MSCs and iMSCs in early and late passages (n = 5). The higher RNA concentration might be related to the increasing cell size of senescent cells. **(B)** Cell cycle analysis was performed by flow cytometry with Propidium Iodide intercalation. The proportion of cells in G2 increase in MSCs of later passage (n = 3), which is in line with previous reports (Gire and Dulić, 2015; Mao et al., 2012). **(C)** For supplementation experiments with orotic acid, we have initially tested the concentration that did not impair viability after three days (Alamar blue assay; n = 3). **(D)** 1 mM orotic acid was subsequently substituted to MSC culture media and there was no consistent impact on long-term growth curves of two MSC preparations (cPD = cumulative population doublings).

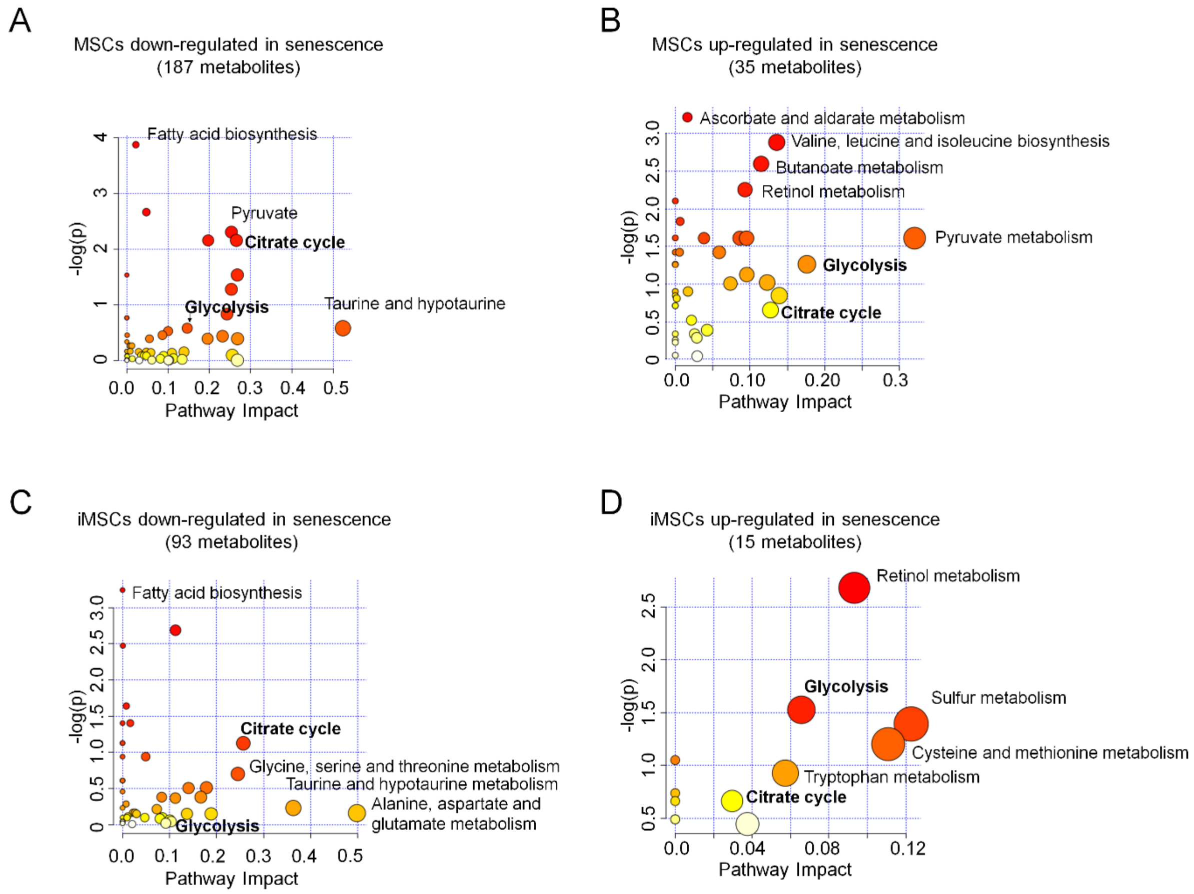

Figure S3: Metabolic Pathway Analysis in either MSC or iMSC, Related to Figure 4.
The metabolic pathway analysis (using MetaboAnalyst 4.0) was performed for metabolites that were either down- or up-regulated during replicative senescence of either MSCs **(A, B)** or iMSCs **(C, D)**. Scores for enrichment (vertical axis) and topology analyses (pathway impact, horizontal axis) are depicted (color code depicts overall significance, and the size of the circles reflects centrality of the involved metabolites).

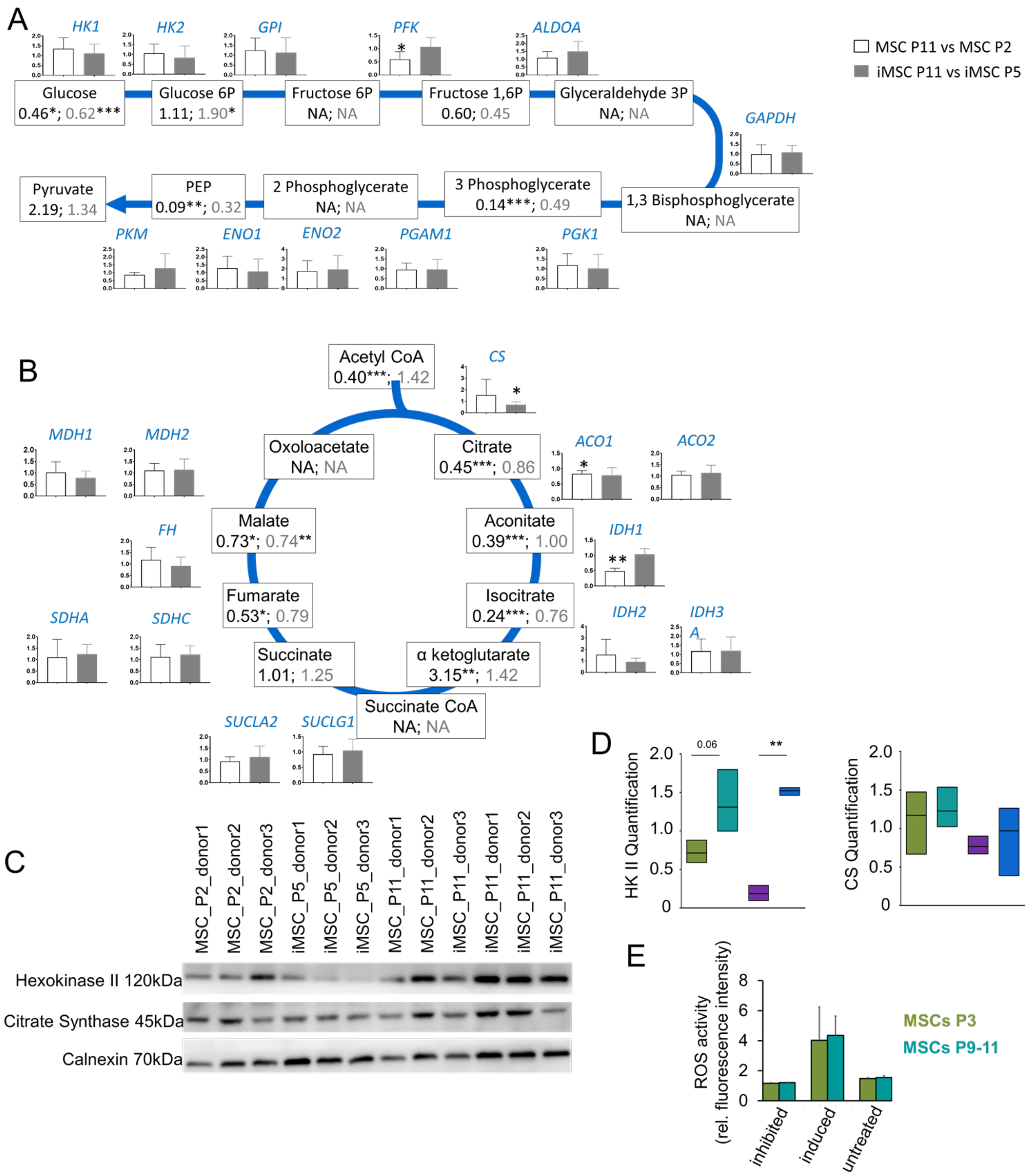

Figure S4: Changes in Glycolysis and Tricarboxylic Acid Cycle, Related to Figure 4.
Senescence-associated gene expression changes of enzymes involved in glycolysis **(A)** and TCA cycle **(B)** are represented by bars (MSCs P11 versus MSCs P2 in white; and iMSCs P11 versus iMSCs P5 in grey). In addition, the corresponding fold-changes of metabolites are indicated in the boxes (for MSCs and iMSCs, respectively). The discrepancies between gene expression and metabolites might be attributed to additional impact on mRNA splicing, translation, posttranslational modification, protein activation and localization. Abbreviations: HK, hexokinase; GPI, glucose-6-phosphate isomerase; PFK, phosphofructokinase; ALDOA, aldolase A; GAPDH, glyceraldehyde 3-phosphate dehydrogenase; PGK1, phosphoglycerate kinase; PGAM1, phosphoglycerate mutase 1; ENO enolase; PKM, pyruvate kinase; CS, citrate synthase; ACO, aconitase; FH, fumarate hydratase; IDH, isocitrate dehydrogenase; MDH,

malate dehydrogenase; SUCL, succinate/malate CoA ligase; SDH, succinate dehydrogenase; NA, not available. **(C)** Western Blots depict exemplarily for two key proteins in Glycolysis (Hexokinase II) and TCA (Citrate Synthase) as well as the housekeeping (Calnexin). **(D)** Relative quantification of Western Blots (n = 3; two technical replica each) reveals increased Hexokinase II (HK II) levels in late passages for MSCs and iMSCs, while no changes for Citrate Synthase (CS) levels. **(E)** Cellular reactive oxygen levels (ROS) demonstrated a slight increase of ROS levels at later passages – despite the metabolic shift – which is in line with previous publications (Geißler et al., 2012; Jeong and Cho, 2015). Fluorescence intensity (488/520 nm) of ROS detection reagent was measured for three different MSC donors at early and late passage (six technical replica for each donor). ROS was inhibited with N-acetyl-L-cysteine and induced using Pyocyanin following the manufacturer´s instructions. The untreated cells did not reveal a clear increase in ROS levels at later passages. In analogy we analyzed superoxide levels, but we did not observe changes in superoxide levels in late passages (data not shown). All results were normalized to unstained cells.

Supplemental Tables

Supplemental Table S1: Differentially Expressed Transcripts upon Senescence of MSCs and iMSCs, Related to Figure 2.
This table is provided as separate EXCEL File

Supplemental Table S2: Identified Metabolites, Related to Figure 3.
This table is provided as separate EXCEL File

Supplemental Methods

Flow Cytometry

Immunophenotypic surface marker analysis was performed on a FACS Canto II (BD, Heidelberg, Germany) upon staining with CD14 allophycocyanin (APC, clone M5E2; BD), CD29 phycoerythrin (PE, clone MAR4; BD), CD31 PE (clone WM59; BD), CD34 APC (clone 8G12; BD), CD45 APC (clone HI30; BD), CD73 PE (clone AD2; BD), CD90 APC (clone 5E10; BD), CD105 fluorescein isothiocyanate (FITC, clone MEM-226; ImmunoTools, Friesoythe, Germany). Autofluorescence was used as negative control.

Analysis of Proliferation

At every passage cells were harvested by trypsinization, counted in a Neubauer cell chamber and seeded in defined numbers. Cumulative population doublings (cPDs) were calculated as described before (Cholewa et al., 2011).

In vitro differentiation of MSCs and iMSCs

Differentiation of MSCs and iMSCs toward adipogenic, osteogenic, and chrondrogenic lineage was induced as previously described (Frobel et al., 2014). After three weeks, osteogenic differentiation of MSCs and iMSCs was analyzed by staining of calcium precipitates with Alizarin Red S and quantified on a Tecan Infinite 200 plate reader (Gregory et al., 2004). Fat droplets in adipogenic differentiation were stained with BODIPY counterstained with DAPI. Glycosaminoglycan deposition in chondrogenic differentiation was analyzed by Alcian Blue and PAS staining.

Transcriptomics

RNA was isolated from the samples using the NucleoSpin RNA extraction kit (Macherey-Nagel, Düren, Germany). Library preparation was performed with the Illumina TruSeq Stranded Total RNA Sample Preparation Kit, with a total input amount of 1 µg, following the standard protocol with ribosomal depletion (Illumina Ribo-Zero Gold rRNA removal kit) and the NextSeq 500/550 High Output Kit v2 (carried out by the Core Genomics-facility IZKF, RWTH Aachen University). The resulting pair-end reads were quality-checked with FastQC (http://www.bioinformatics.babraham.ac.uk/projects/fastqc/), and low-quality reads were removed using TrimGalore. Reads were mapped to the GRCh38 assembly of the human genome using Tophat2 (Trapnell et al., 2009), version 2.0.10, and reassembled with Cufflinks (Trapnell et al., 2012), version 2.1.1. Differential gene expression was analyzed using the DESeq2 package (Love et al., 2014), version 1.10.1. Significance of transcriptomic changes was estimated using the R limma package with an adjusted paired p-value <0.05. Gene Ontology analysis was performed with GoMiner™.

Immunoblot Analysis

For protein isolation, 100 µl of RIPA lysis buffer (50 mM Tris-HCL pH 7.5, 150 mM NaCl 1 mM EDTA, 0.1 % sodium deoxycholate, 1 % NP-40, 1x protease inhibitor (Sigma) and 1 x phosphatase inhibitor (Roche)) was added to 1 million cells. Protein lysates were transferred to 1.5 ml Eppendorf tubes and snap-frozen in liquid nitrogen and thawed on ice for three repeated cycles. After 10 min of centrifugation at 12,000 g and 4° C the supernatant of all samples was collected into fresh tubes and and protein concentration was determined using the Bradford colorimetric protein method (Thermo Fisher Scientific). Protein samples were stored at -80 °C. Samples for immunoblotting were separated by SDS-PAGE after being prepared with 4x Laemmli Sample Buffer containing 10 % β-mercaptoethanol and heated to 95° C for 5 min. Subsequently, proteins were transferred to nitrocellulose membrane and blocked with Western Blocking Reagent (Roche). Membranes were incubated with primary antibodies at 4° C overnight. After incubation with secondary antibodies, blots were developed with Pierce ECL Western Blotting substrate (Thermo Fisher Scientific). All densitometry measurements were performed with ImageJ. Band intensity was normalized to calnexin (CLNX), which served as internal loading control, blotted on the same gel and membrane. Citrate synthase antibody was purchased from Cell Signaling Technology (catalogue no. 14309), while hexokinase II antibody was purchased from Abcam (catalogue no. (ab131196). Primary antibodies were used at a 1:1000 dilution ratio in Tween TBS. Anti-CLNX antibody was used at a 1:5,000 dilution and purchased from Calbiochem (catalogue no. 208-880).

Quantification of orotic acid

A confluent well of a six-well plate was used to isolate the metabolites with methanol (Ser et al., 2015), while another well was used to determine the total protein concentration to normalize the results . Briefly, 50 µL of the sample was mixed with 50 µL of 4.0 mM of 4-TFMBAO, 50 µL of 8.0 mM $K_3[Fe(CN)_6]$ and 50 µL of 80 mM K_2CO_3. The mixture (200 µL) was then heated at 80 °C for 4 min, followed by cooling in an ice bath for approx. 2 min to stop the reaction. The relative fluorescence intensity produced by the reaction with 4-TFMBAO was measured with TECAN 200 plate reader (Tecan Group Ltd., Switzerland) at maximum excitation and emission wavelengths of 340 and 460 nm, respectively.

Metabolic flux analysis

The XFe96 Flux analyzer and Prep Station (Seahorse Bioscience XFe96 Instrument, Agilent, CA, USA) were used according to the manufacturer's instructions (Ferrick et al., 2008). Briefly, cells were seeded in XFe96 cell culture plates at 25,000 cells per well and cultured overnight. The XFe96 sensor cartridges were hydrated overnight with 200 µL of Seahorse Bioscience XFe96 Calibrant at pH 7.4 and stored at 37 °C without CO_2. One hour before the measurement, cells were washed and culture media were replaced with no-glucose media. Basal measurements of ECAR and OCR, as well as measurements after addition of glucose (final concentration 10 mM; Sigma Aldrich, St. Louis, MO, USA), oligomycin (final

concentration 5 µM; Sigma Aldrich) and 2-deoxy-d-glucose (2DG, final concentration 100 mM; Seahorse Bioscience), were performed as described in the XF Glycolysis Stress Test Kit User Manual (Seahorse Bioscience).

Cellular ROS/Superoxide Detection Assay Kit

Oxidative stress production was investigated through Cellular ROS/Superoxide detection assay kit (ab139476, Abcam, Cambridge, UK) following the manufacturer's protocol. Briefly, MSCs and iMSCs cells were seeded (1×10^4 cells/well) into transparent 96-well plates and maintained to attach at 37 °C in 5% CO2. After 24 h cells were pre-incubated with superoxide detection reagent (orange) or ROS detection reagent (green) for 1 h. After that, the fluorescence intensity was measured with a Tecan Infinite 200 plate reader (Tecan Group Ltd., Männedorf, Switzerland), with an excitation and emission wavelengths of 550/610 nm and for Superoxide detection and 488/520 nm to detect ROS.

Supplemental References

Cholewa, D., Stiehl, T., Schellenberg, A., Bokermann, G., Joussen, S., Koch, C., Walenda, T., Pallua, N., Marciniak-Czochra, A., Suschek, C. V, et al. (2011). Expansion of adipose mesenchymal stromal cells is affected by human platelet lysate and plating density. Cell Transplant. *20*, 1409–1422.

Delfarah, A., Parrish, S., Junge, J.A., Yang, J., Seo, F., Li, S., Mac, J., Wang, P., Fraser, S.E., and Graham, N.A. (2019). Inhibition of nucleotide synthesis promotes replicative senescence of human mammary epithelial cells. J. Biol. Chem. *294*, 10564–10578.

Ferrick, D.A., Neilson, A., and Beeson, C. (2008). Advances in measuring cellular bioenergetics using extracellular flux. Drug Discov. Today *13*, 268–274.

Frobel, J., Hemeda, H., Lenz, M., Abagnale, G., Joussen, S., Denecke, B., Sarić, T., Zenke, M., and Wagner, W. (2014). Epigenetic rejuvenation of mesenchymal stromal cells derived from induced pluripotent stem cells. Stem Cell Reports *3*, 414–422.

Geißler, S., Textor, M., Kühnisch, J., Könnig, D., Klein, O., Ode, A., Pfitzner, T., Adjaye, J., Kasper, G., and Duda, G.N. (2012). Functional Comparison of Chronological and In Vitro Aging: Differential Role of the Cytoskeleton and Mitochondria in Mesenchymal Stromal Cells. PLoS One *7*, e52700.

Gire, V., and Dulić, V. (2015). Senescence from G2 arrest, revisited. Cell Cycle *14*, 297–304.

Gregory, C.A., Gunn, W.G., Peister, A., and Prockop, D.J. (2004). An Alizarin red-based assay of mineralization by adherent cells in culture: comparison with cetylpyridinium chloride extraction. Anal. Biochem. *329*, 77–84.

Hernandez-Segura, A., de Jong, T. V., Melov, S., Guryev, V., Campisi, J., and Demaria, M. (2017). Unmasking Transcriptional Heterogeneity in Senescent Cells. Curr. Biol. *27*, 2652-2660.e4.

Jeong, S.-G., and Cho, G.-W. (2015). Endogenous ROS levels are increased in replicative senescence in human bone marrow mesenchymal stromal cells. Biochem. Biophys. Res. Commun. *460*, 971–976.

Love, M.I., Huber, W., and Anders, S. (2014). Moderated estimation of fold change and dispersion for RNA-seq data with DESeq2. Genome Biol. *15*, 550.

Mao, Z., Ke, Z., Gorbunova, V., and Seluanov, A. (2012). Replicatively senescent cells are arrested in G1 and G2 phases. Aging (Albany. NY). *4*, 431–435.

Ser, Z., Liu, X., Tang, N.N., and Locasale, J.W. (2015). Extraction parameters for metabolomics from cultured cells. Anal. Biochem. *475*, 22–28.

Trapnell, C., Pachter, L., and Salzberg, S.L. (2009). TopHat: discovering splice junctions with RNA-Seq. Bioinformatics *25*, 1105–1111.

Trapnell, C., Roberts, A., Goff, L., Pertea, G., Kim, D., Kelley, D.R., Pimentel, H., Salzberg, S.L., Rinn, J.L., and Pachter, L. (2012). Differential gene and transcript expression analysis of RNA-seq experiments with TopHat and Cufflinks. Nat. Protoc. *7*, 562–578.
