# Supplementary material for: Senescence-Associated Metabolomic Phenotype in Primary and iPSC-Derived Mesenchymal Stromal Cells
Source: Stem Cell Reports. 2020 Jan 23;14(2):201–9. doi: 10.1016/j.stemcr.2019.12.012 (PMC7013233; doi:10.1016/j.stemcr.2019.12.012)
Supplement: Document S2. Article plus Supplemental Information [file mmc4.pdf]

Stem Cell Reports
Report
OPEN ACCESS

Senescence-Associated Metabolomic Phenotype in Primary and iPSC-Derived Mesenchymal Stromal Cells

Eduardo Fernandez-Rebollo,[1,2,6,*] Julia Franzen,[1,2] Roman Goetzke,[1,2] Jonathan Hollmann,[1,2] Alina Ostrowska,[1,2] Matteo Oliverio,[3,4] Torsten Sieben,[1,2] Björn Rath,[5] Jan-Wilhelm Kornfeld,[3,4,6] and Wolfgang Wagner[1,2,*]

[1]Helmholtz-Institute for Biomedical Engineering, Stem Cell Biology and Cellular Engineering, RWTH Aachen University Medical School, Aachen 52074, Germany
[2]Institute for Biomedical Technology – Cell Biology, RWTH Aachen University Medical School, Aachen 52074, Germany
[3]Max Planck Institute for Metabolism Research (MPI-MR), Noncoding RNAs and Energy Homeostasis, Gleueler Strasse 50, Cologne 50931, Germany
[4]Cologne Cluster of Excellence: Cellular Stress Responses in Aging-Associated Diseases (CECAD), Cologne 50931, Germany
[5]Department for Orthopedics, RWTH Aachen University Medical School, Aachen 52074, Germany
[6]University of Southern Denmark, Functional Genomics and Metabolism Unit, Department for Biochemistry and Molecular Biology, Campusvej 55, Odense 5230, Denmark
*Correspondence: ef-r@bmb.sdu.dk (E.F.-R.), wwagner@ukaachen.de (W.W.)
https://doi.org/10.1016/j.stemcr.2019.12.012

SUMMARY

Long-term culture of primary cells is characterized by functional and secretory changes, which ultimately result in replicative senescence. It is largely unclear how the metabolome of cells changes during replicative senescence and if such changes are consistent across different cell types. We have directly compared culture expansion of primary mesenchymal stromal cells (MSCs) and induced pluripotent stem cell-derived MSCs (iMSCs) until they reached growth arrest. Both cell types acquired similar changes in morphology, *in vitro* differentiation potential, senescence-associated β-galactosidase, and DNA methylation. Furthermore, MSCs and iMSCs revealed overlapping gene expression changes, particularly in functional categories related to metabolic processes. We subsequently compared the metabolomes of MSCs and iMSCs and observed overlapping senescence-associated changes in both cell types, including downregulation of nicotinamide ribonucleotide and upregulation of orotic acid. Taken together, replicative senescence is associated with a highly reproducible senescence-associated metabolomics phenotype, which may be used to monitor the state of cellular aging.

INTRODUCTION

In vitro culture of primary cells is associated with continuous changes that ultimately result in replicative senescence: the proliferation rate declines, cells enlarge, and they lose differentiation potential (Campisi and d'Adda di Fagagna, 2007). These profound changes in the course of culture expansion hamper the reproducibility of experiments, which is of particular relevance in regenerative medicine (Wagner and Ho, 2007). For example, mesenchymal stromal cells (MSCs) acquire continuous changes in gene expression and DNA methylation over subsequent passages, which can be used to track the state of cellular aging (Koch et al., 2012; Li et al., 2017; Schellenberg et al., 2014; Wagner et al., 2008). In contrast to quiescent or apoptotic cells, senescent cells are highly metabolically active (Wiley and Campisi, 2016). Senescent cells secrete a characteristic cocktail of interleukins, chemokines, and growth and inflammatory factors, which compose the senescence-associated secretory phenotype (SASP) (Coppé et al., 2008). The SASP was meanwhile observed across multiple cell types, and there is evidence that this secretory function of senescent cells has an impact on wound healing, embryonic development, and tumorigenesis (Watanabe et al., 2017). However, little is known about the metabolomic changes that may be associated with or even contribute to the process of cellular aging.

In contrast to primary cells, such as MSCs, induced pluripotent stem cells (iPSCs) do not reveal any signs of replicative senescence (Koch et al., 2013; Lapasset et al., 2011). They can be culture expanded for many passages without reduced proliferation, loss of differentiation potential, or telomere attrition. It is unclear how iPSCs escape from replicative senescence, while there is evidence that their progeny are bound to this destiny again upon exit from the pluripotent state (Frobel et al., 2014). Long-term growth curves and the molecular sequel of replicative senescence have so far hardly been systematically addressed in iPSC-derived cells. A better understanding of cellular aging upon differentiation of iPSCs is therefore urgently needed with regard to the enormous hopes for regenerative medicine.

Various protocols have been described for generating iPSC-derived MSC-like cells (iMSCs) (Diederichs and Tuan, 2014; Frobel et al., 2014; Kang et al., 2015). These cells closely resemble their primary counterparts in morphology, immunophenotype, and three-lineage differentiation potential toward osteocytes, chondrocytes, and adipocytes (Sabapathy and Kumar, 2016). It has been

This is an open access article under the CC BY license (http://creativecommons.org/licenses/by/4.0/).

suggested that iMSCs might be more homogeneous than primary MSCs, which are well known to comprise multiple subpopulations (Ho et al., 2008). However, on the epigenetic level MSCs and iMSCs still remained distinct and DNA methylation patterns that reflect the tissue of origin or donor age were not recapitulated in iMSCs (Frobel et al., 2014). So far, it was unclear whether iMSCs undergo the same molecular changes as primary MSCs during culture expansion. In this study, we have therefore compared functional, transcriptomic, and metabolomic changes during long-term growth of MSCs and iMSCs.

RESULTS

iPSC-Derived Mesenchymal Stromal Cells Are Bound to Replicative Senescence

MSCs at first passage (n = 5) were reprogrammed into iPSCs and then redifferentiated toward MSCs (iMSCs). Syngeneic MSCs and iMSCs were subsequently expanded until the cells entered proliferation arrest. MSC proliferation rates decreased after about 20–40 days, whereas iMSCs proliferated more slowly, with a later decline in proliferation rate. Within 3 months all cell populations reached the senescent state, with a mean number of population doublings of 21.3 ± 1.4 and 17.1 ± 3.8 for MSCs and iMSCs, respectively (Figure 1A). The changes in cellular morphology were very comparable between MSCs and iMSCs: at early passages they displayed spindle-shaped fibroblast-like morphology, whereas cells at later passages were enlarged, with flattened “fried egg” morphology (Figure 1B).

The immunophenotypes of MSCs and iMSCs were similar ($CD29^+$, $CD73^+$, $CD90^+$ $CD105^+$, $CD14^-$, $CD31^-$, $CD34^-$, and $CD45^-$) and remained stable throughout culturing (Figure S1A). MSCs and iMSCs of early passages could be differentiated *in vitro* toward osteogenic, adipogenic, and chondrogenic lineages (Frobel et al., 2014). The differentiation potential of MSCs decayed at later passages, as described before (Wagner et al., 2008), and this was also observed for iMSCs (Figure S1B).

Both cell types revealed increased staining for senescence-associated β-galactosidase (SA-β-gal) at later passages, albeit several iPSCs and iMSCs at early passages also stained positive for SA-β-gal (Figure 1C). Subsequently, we estimated the state of cellular aging based on epigenetic modifications. We previously demonstrated that the number of passages is reflected by concurring DNA methylation changes at six senescence-associated CG dinucleotides (Franzen et al., 2017; Koch et al., 2012). MSCs and iMSCs revealed increasing epigenetic senescence predictions at later passages, whereas iPSCs were even predicted to be of negative passages (Figure 1D). Taken together, iMSCs fulfilled the minimal criteria for definition of MSCs (Dominici et al., 2006), and both cell types apparently reveal very similar modifications during long-term culture that ultimately result in replicative senescence.

Senescence-Associated Gene Expression Is Closely Related in MSCs and iMSCs

It has been demonstrated that long-term culture of MSCs is reflected by unique changes in their gene expression profiles (Wagner et al., 2008). To address the question of whether iMSCs reflect similar modifications we compared the transcriptomes of MSCs and iMSCs at early and late passages by deep sequencing (n = 5). Overall the gene expression profiles of MSCs and iMSCs were closely related, albeit they were clearly separated by hierarchical clustering regarding cell types and passages (Figure 2A).

Pairwise comparison of MSCs and iMSCs revealed significant differences between early and late passages (2-fold differential expression and adjusted $p < 0.05$): 613 and 563 transcripts were differentially expressed upon senescence of MSCs and iMSCs, respectively (Figure 2B; Table S1). We did not observe consistent upregulation of SASP-associated genes (Figure S1C). Notably, senescence-associated changes in MSCs and iMSCs share a relatively high number of downregulated transcripts (61 transcripts; hypergeometric distribution: $p < 10^{-17}$) and upregulated transcripts (32 transcripts; $p < 10^{-9}$; Figure 2C). Senescence-associated gene expression changes were particularly enriched in Gene Ontology (GO) categories related to metabolism, such as cellular metabolic process, nitrogen compound metabolic process, or organic substance metabolic process, among others (Figure 2D). Genes associated with the GO category metabolic process (GO: 0008152) are highlighted in Table S1. These results indicate that metabolic changes might be of particular relevance for the process of cellular aging in MSCs and iMSCs.

Senescence Is Reflected by Consistent Changes in the Metabolome

To gain insight into how the metabolism varies during long-term culture, we analyzed the metabolomes of MSCs at passage 2 (n = 5) and passage 11 (n = 5) and of iMSCs at passage 5 (n = 5) and passage 11 (n = 5). For comparison we also analyzed the five corresponding iPSC preparations. Using chromatographic approaches (ultra-high-performance liquid chromatography/mass spectrometry/mass spectrometry [UHPLC/MS/MS] and gas chromatography/mass spectrometry [GC/MS]), 612 different metabolites were identified and measured (Table S2). Hierarchical clustering of metabolomic profiles clearly separated cell types as well as early and late passages. In fact, MSCs and iMSCs at passage 11 comprised similar metabolites (Figure 3A).

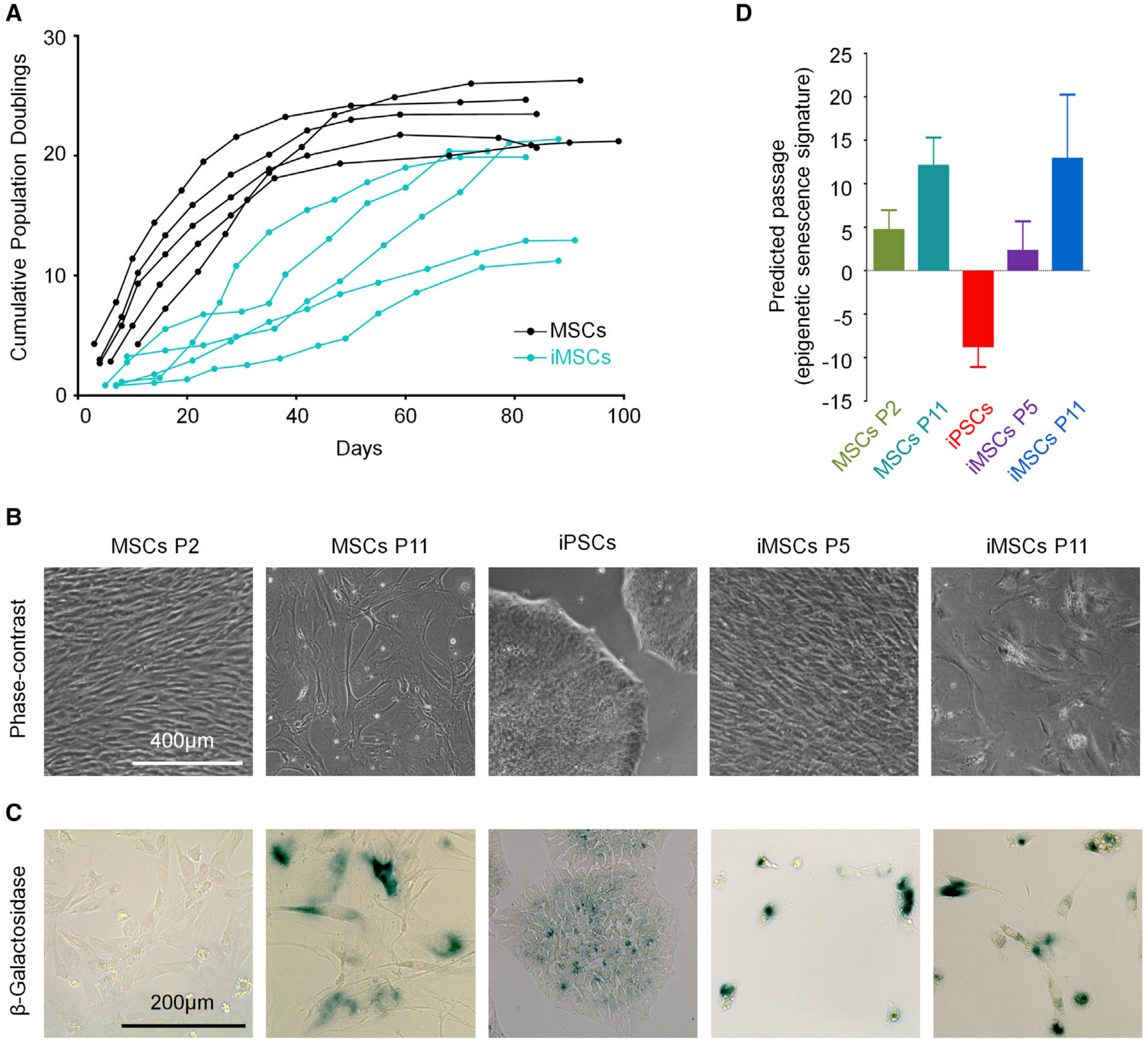

Figure 1. Replicative Senescence of iPSC-derived MSCs

(A) Long-term growth curves of syngeneic MSCs and iMSCs demonstrate that both cell types enter proliferative arrest within 3 months.

(B) Phase-contrast images of MSCs (passages 2 and 11) and iPSCs and iMSCs (passages 5 and 11).

(C) Staining for senescence-associated β-galactosidase was more pronounced at later passages, albeit several iPSCs and iMSCs at early passage were also detected positive.

(D) The number of passages was estimated based on an epigenetic senescence signature that utilizes DNA methylation levels at six CG dinucleotides (n = 5 biological replicates) (Koch et al., 2012).

We subsequently analyzed if there were significant differences in the metabolomic profiles of cells at early and late passages (>1.5-fold difference and adjusted $p < 0.05$): 222 and 108 metabolites were significantly changed in MSCs and iMSCs, respectively (Figure 3B; Table S2). Notably, there was a highly significant overlap of senescence-associated metabolic changes in MSCs and iMSCs: 67 metabolites were consistently downregulated (hypergeometric distribution: $p < 10^{-43}$) and 9 metabolites were upregulated at later passages in both cell types ($p < 10^{-10}$; Figure 3C). In contrast, only sphingomyelin was significantly upregulated during senescence of MSCs and downregulated in iMSCs, while inosine and uridine metabolites were upregulated during senescence of iMSCs and downregulated in MSCs. These results demonstrate that long-term culture of MSCs and iMSCs is overall associated with similar metabolomic changes. Among the metabolites with the most drastic downregulation in senescence were N-acetylglucosamine 6-phosphate, N-acetylglucosamine 1-phosphate, nicotinamide riboside, and nicotinamide ribonucleotide, whereas upregulated metabolites included orotate, gamma-glutamylphenylalanine, 4-hydroxybutyrate, and

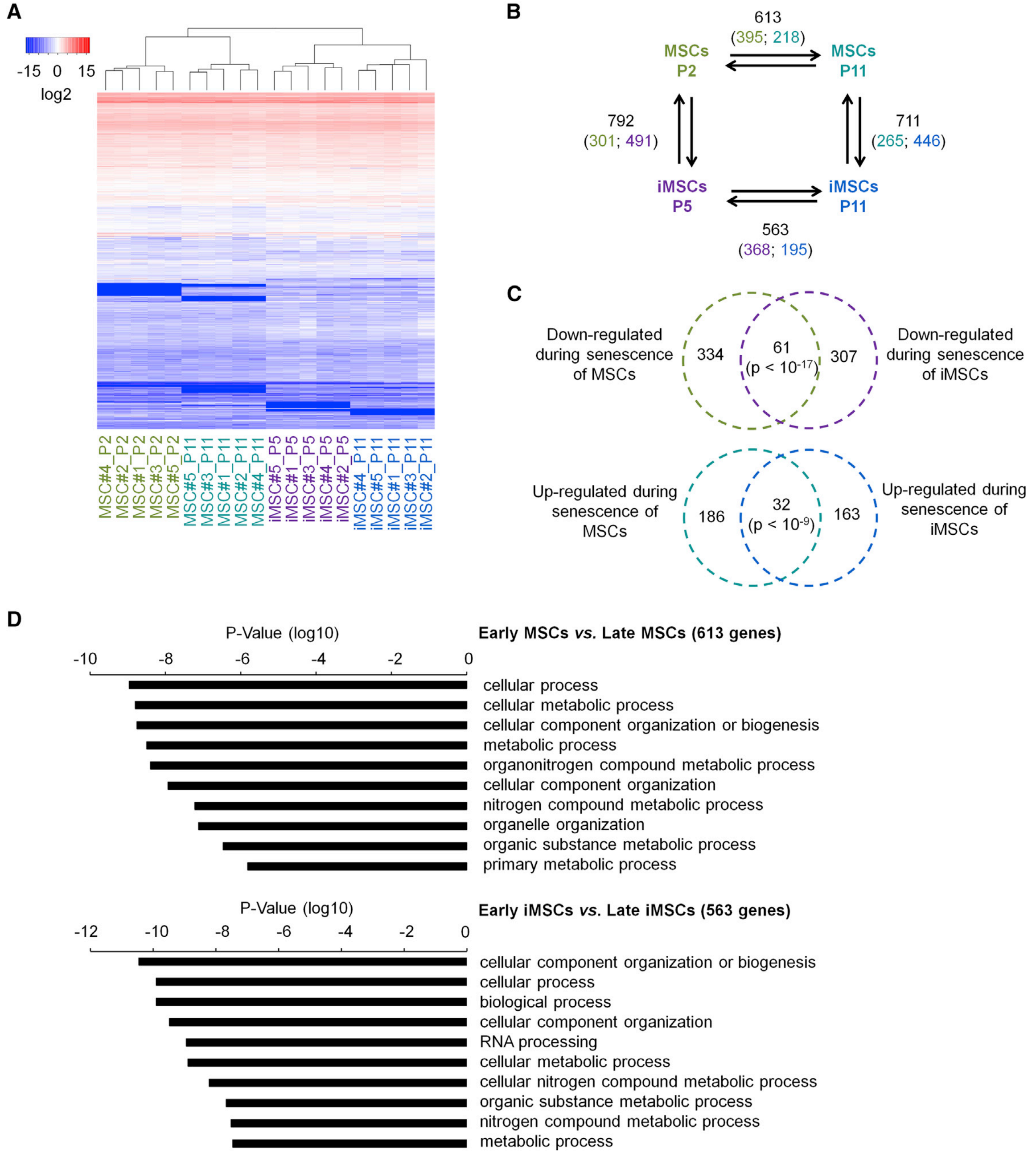

Figure 2. Transcriptome Analysis of Replicative Senescence

(A) Heatmap of the entire transcriptome of MSCs and corresponding iMSCs (n = 5, donor numbers are indicated). Hierarchical clustering clearly separated early and late passages for both cell types.

(B) The numbers of transcripts with significant gene expression changes in pairwise comparisons are depicted (2-fold differential expression and limma adjusted $p < 0.05$; the number of upregulated genes in the corresponding cell type is indicated by color code).

(C) The Venn diagrams depict significant overlaps of down- and upregulated transcripts during senescence of MSCs and iMSCs (significance was estimated by hypergeometric distribution).

(D) The senescence-associated gene expression changes in MSCs and iMSCs were classified by Gene Ontology analysis. Functional categories involved in metabolic processes were among the most significantly overrepresented (Fisher t test).

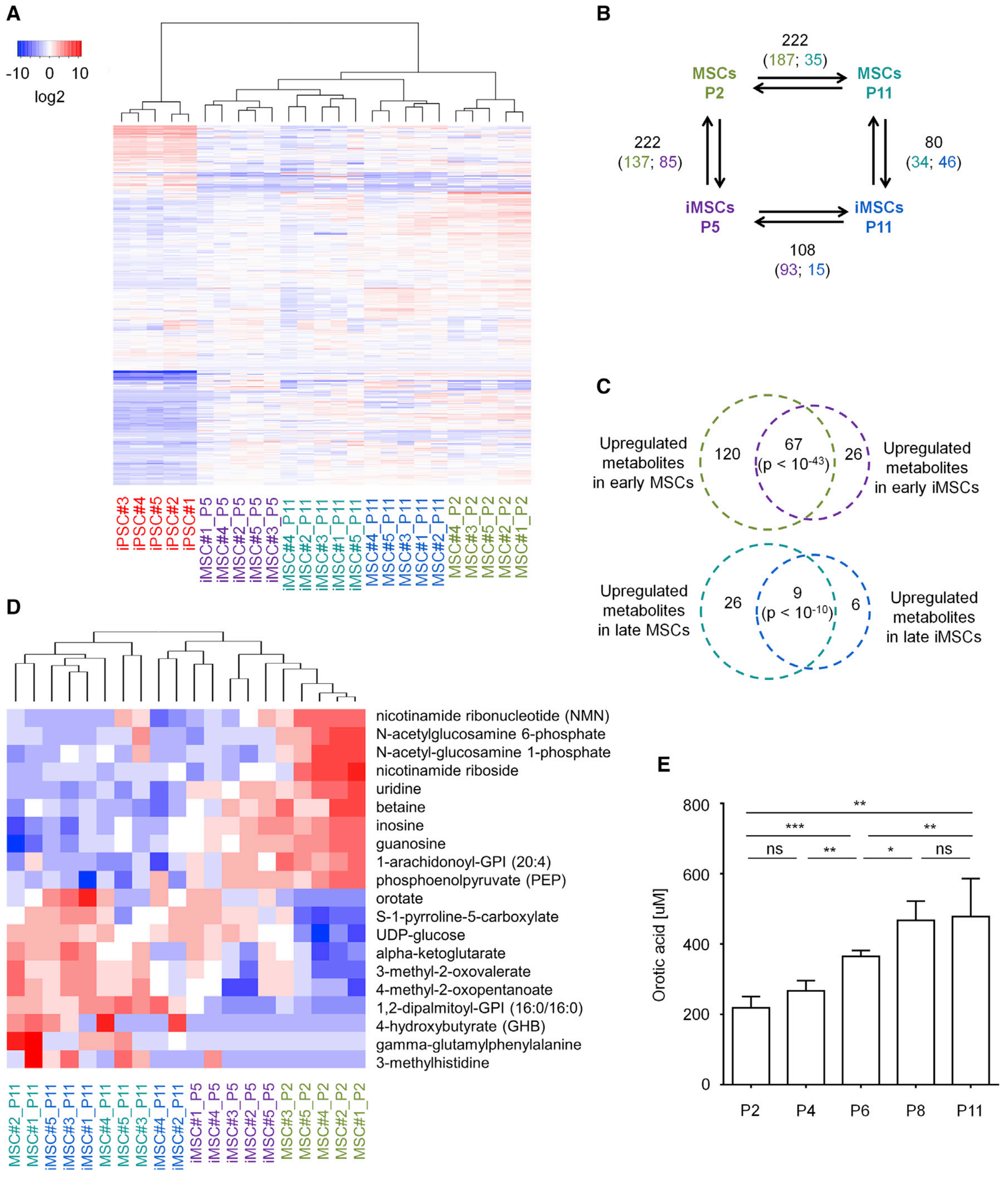

Figure 3. Metabolomic Characterization of Replicative Senescence

(A) Heatmap presentation of metabolomes (612 different metabolites). Hierarchical clustering clearly separated iPSCs, MSCs, and iMSCs, as well as cells at early and late passage (n = 5, donor numbers are indicated).

(B) Schematic presentation of the numbers of metabolites with significant differences in pairwise comparisons (1.5-fold difference and adjusted $p < 0.05$; higher abundance is indicated by the color code of corresponding cell type).

(C) The Venn diagrams depict a high overlap of senescence-associated changes in MSCs and iMSCs for upregulated and downregulated metabolites (significance was estimated by hypergeometric distribution).

(D) Heatmap of the ten most significant up- and downregulated metabolites during replicative senescence (MSCs + iMSCs).

(E) Orotic acid was exemplarily measured by a fluorogenic assay in MSCs (n = 4 biological replicates) and increased over subsequent passages, which is in line with our metabolomics results. * p. < 0.05; ** $p < 0.005$ and *** $p < 0.001$.

3-methylhistidine (Figure 3D). Taken together, replicative senescence is associated with significant metabolomic changes that are similar in MSCs and iMSCs.

We have exemplarily validated the senescence-associated changes in orotic acid in cell pellets of MSCs (n = 4) using a fluorogenic reaction (Yin et al., 2015). The results validated a significant increase in orotic acid over subsequent passages (Figure 3E). Orotic acid is needed for pyrimidine synthesis and, thus, the increase might result from DNA/RNA synthesis abrogation. However, the RNA concentration rather increased in later passages, and senescent cells were often stalled in G2 (Figures S2A and S2B). To further analyze if increasing concentrations of orotic acid might be functionally relevant for senescence, we performed additional long-term culture experiments with culture medium that was supplemented with 1 mM orotic acid, but there was no consistent acceleration of replicative senescence (Figures S2C and S2D). While these results indicate that orotic acid is not functionally relevant for the process of cellular senescence, the quantification of specific metabolites can be used to track cellular aging.

The Bioenergetic Switch in Senescence

To gain better insight into how the senescence-associated metabolites map to specific metabolic pathways, we used MetaboAnalyst (Xia et al., 2015). The identified pathways were similar for MSCs and iMSCs and even had a similar enrichment for up- and downregulated metabolites, which may indicate that the relevant proteins of these pathways are regulated independently (Figure S3). For simplicity, we subsequently focused on all metabolites that were at least significantly changed in MSCs and/or iMSCs: 213 downregulated metabolites were particularly associated with the glycerophospholipid pathway ($p = 2.6 \times 10^{-7}$ and pathway impact score [PI] = 0.47) and the tricarboxylic acid cycle ($p = 9.2 \times 10^{-6}$ and PI = 0.27), as well as the taurine and hypotaurine pathways (p = 0.0084 and PI = 0.52; Figure 4A). On the other hand, the 41 upregulated metabolites were enriched in the biosynthesis of valine, leucine, and isoleucine (p = 0.0019 and PI = 0.14), butanoate metabolism (p = 0.0060 and PI = 0.11), pyruvate metabolism (p = 0.0083 and PI = 0.017), and glycolysis (p = 0.015 and PI = 0.18; Figure 4B). These results indicated that changes in the tricarboxylic acid cycle and glycolysis might be of particular relevance during senescence. Overall, these pathways seemed to be hardly regulated on the gene expression level (Figures S4A and S4B), but on the protein level, we observed a significant upregulation of hexokinase II at late passages of MSCs and iMSCs, which provides additional evidence for the enhanced glycolytic state (Figure S4C).

In fact, previous studies indicated that various cell types gradually shift toward a more glycolytic state during culture expansion (Bittles and Harper, 1984; Zwerschke et al., 2003). We therefore analyzed if this metabolic switch occurs also in MSCs and iMSCs using a flux analyzer to monitor the extracellular acidification rate (ECAR) and the mitochondrial oxygen consumption rate (OCR). ECAR rather reflects glycolytic behavior and it was consistently higher in senescent MSCs and iMSCs (Figure 4C). In contrast, OCR was higher and more affected in early passages of MSCs and iMSCs (Figure 4D). Since mitochondrial function might be impaired, we also analyzed the amount of reactive oxygen species (ROS), which are by-products of the respiratory chain. We observed only a very moderate upregulation of ROS in later passages (Figure S4D). Taken together, MSCs and iMSCs reveal the same senescence-associated shift to glycolytic pathways upon senescence.

DISCUSSION

Recently, the first clinical trials with iMSCs were started for steroid-refractory graft versus host disease (Rasko et al., 2019), and it will be important to understand if these cell preparations are equally as safe and potent as primary MSCs. In this context, it is also crucial to better understand how culture expansion affects iMSCs. The results of this study indicate that upon exit from the pluripotent state the iMSCs undergo the same functional and molecular changes as previously described for primary MSCs: iMSCs recapitulate senescence-associated changes in morphology, loss of differentiation potential, and ultimately growth arrest. Furthermore, the senescence markers SA-β-gal and the epigenetic senescence signature increased with serial passaging. It might have been anticipated that iMSCs, which are derived from entirely rejuvenated iPSCs, could reach a higher number of cumulative population doublings, whereas our results indicate that iMSCs enter the senescent state after fewer cumulative population doublings even than primary MSCs. On the other hand, the state of cellular aging may be better controlled in iPSC-derived cells by the possibility of expanding iPSCs in the pluripotent state to then generate iMSCs of low passage in relevant cell numbers.

Only a few studies have investigated metabolomic changes during culture expansion, and most of them addressed changes in fibroblasts (James et al., 2015; Zwerschke et al., 2003). Overall, there was a consistent shift from oxidative phosphorylation toward glycolysis and the pentose phosphate pathway (James et al., 2016; Zwerschke et al., 2003). The metabolic switch might be relevant to counteract extensive accumulation of ROS levels. The increase in these reactive intermediates might be attributed to the finding that mitochondria at later passages are often dysfunctional (Zhang et al., 2018). An alternative

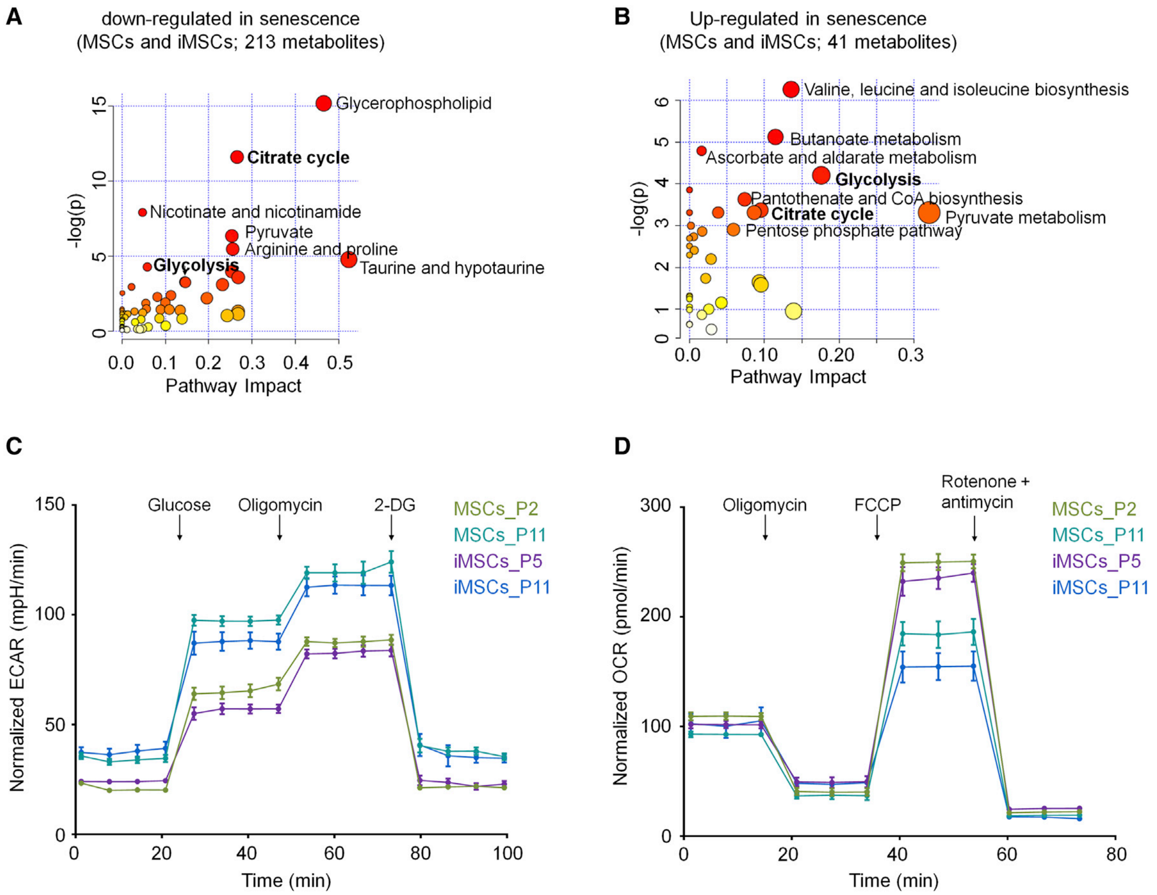

Figure 4. Metabolic Pathway Analysis during Replicative Senescence

(A and B) (A) The metabolic pathway analysis (using MetaboAnalyst 4.0) indicated that the 213 metabolites that were significantly downregulated during senescence of MSCs and/or iMSCs pointed toward more oxidative metabolism, whereas (B) the 41 upregulated metabolites were rather related to glycolytic metabolism. Scores for enrichment (vertical axis) and topology analyses (pathway impact, horizontal axis) are depicted (color code depicts overall significance, and the size of the circles reflects centrality of the involved metabolites).

(C and D) (C) Metabolic flux analysis (Seahorse Bioscience) of the extracellular acidification rate (ECAR) indicates that senescent MSCs and iMSCs use more glycolysis, while (D) the oxygen consumption (OCR) indicates higher oxidative metabolism in MSCs and iMSCs of early passages (n = 3 biological replicates and n = 8 technical replicas for each condition).

explanation for the metabolic shift might be a vicious cycle, where depletion of ATP leads to an adaptive response that increases metabolic imbalance (Zwerschke et al., 2003).

Our results demonstrate that replicative senescence has a major impact on the metabolome, with highly reproducible changes across different cell types and biological replica. Lee et al. have previously identified eight metabolites with altered expression during senescence of MSCs, including lysophosphatidylcholine and lysophosphatidylethanolamine (Lee et al., 2014), while we describe a multitude of significant changes. At this point, it is unclear if the senescence-associated metabolic changes are functionally relevant *per se* or rather a consequence of other modifications, e.g., on chromatin or the transcriptome level. At least for orotic acid, we did not observe a clear impact on the senescence process. Either way, the highly consistent changes indicate that the metabolic phenotype can be used as a biomarker to assess the state of cellular aging.

EXPERIMENTAL PROCEDURES

Cell Culture of Mesenchymal Stromal Cells

MSCs were isolated from the bone marrow of five donors (ranging from 50 to 74 years of age) after orthopedic hip replacement surgery. All samples were taken after written consent according to the guidelines of the local ethics committees (RWTH Aachen; EK300/13) and isolated as described before (Koch et al., 2013). In

brief, cells were flushed from the bone and cultured in parallel in basal medium consisting of Dulbecco's modified Eagle's medium (1 g/L glucose; PAA, Pasching, Austria), with 1% penicillin/streptomycin (PAA) and 1% L-glutamine, and supplemented with 10% human platelet lysate (HPL). HPL pools consisted of at least five lysates to reduce variation, and coagulation was prevented by 0.61 IU unfractionated heparin (Ratiopharm, Ulm, Germany).

Generation of iPSCs and Differentiation toward MSCs

IPSCs were reprogrammed from the same MSC donors that were used for experiments with primary MSCs. Reprogramming was performed with episomal plasmids (Willmann et al., 2013). iPSCs were cultured on tissue culture plastic coated with vitronectin (0.5 μg/cm^2) in StemMACS iPS-Brew XFe96 (all Miltenyi Biotec, Bergisch Gladbach, Germany). Pluripotency was validated by *in vitro* differentiation and Epi-Pluri-Score (Cygenia, Aachen, Germany) (Lenz et al., 2015). iPSCs were redifferentiated toward iMSCs under MSC culture conditions as described above (Frobel et al., 2014). After 1 week, differentiated iPSCs were maintained on 0.1% gelatin-coated TCP or HPL-gel and passaged every further week using trypsin-EDTA 0.25% (Gibco/Thermo Fisher Scientific).

Metabolomics

Pellets of 3×10^6 cells were subjected to methanol extraction and then split into aliquots for analysis by UHPLC/MS/MS and GC/MS (Tufi et al., 2014). Metabolomics analysis was conducted at Metabolon (Durham, NC, USA) as previously described (Long et al., 2017). Briefly, metabolites were identified by automated comparison of ion features to a reference library of chemical standards followed by visual inspection for quality control. Missing values were assumed to be below the detection limits and imputed with the compound minimum (minimum value imputation). To identify the most relevant metabolic pathways during replicative senescence we employed the pathway enrichment analysis for the up- and downregulated metabolites in MSCs and iMSCs using the pathway analysis tool from MetaboAnalyst 4.0 (www.metaboanalyst.ca). The pathway enrichment analysis used GlobalTest to analyze the concentration values with high sensitivity and to identify subtle changes involved in the same biological pathway (Chong et al., 2018). Statistical tests were performed using the R Bioconductor package and significance was estimated using one-way ANOVA and adjusted $p < 0.05$.

Additional information on flow cytometry, proliferation analysis, *in vitro* differentiation of MSCs and iMSCs, transcriptomics, quantification of orotic acid, western blot, metabolic flux assay, and ROS detection is provided in the Supplemental Information.

SUPPLEMENTAL INFORMATION

Supplemental Information can be found online at https://doi.org/10.1016/j.stemcr.2019.12.012.

AUTHOR CONTRIBUTIONS

E.F.-R. and W.W. developed the study concept and experimental design. E.F.-R., J.F., R.G., J.H., A.O., T.S., and M.O. performed the experiments and interpreted the data. J.-W.K. contributed vital reagents and materials and provided important intellectual support throughout the study. E.F.-R. and W.W. wrote the first draft of the manuscript. All authors read and approved the final manuscript.

CONFLICTS OF INTEREST

W.W. is cofounder of Cygenia GmbH (www.cygenia.com), which may provide service for the epigenetic senescence signature to other scientists. R.G. and J.F. also contribute to this company.

ACKNOWLEDGMENTS

This work was funded by the START-Program (to E.F.-R.) and by the Interdisciplinary Center for Clinical Research (IZKF; O3-3; to W.W.), both within the Faculty of Medicine at the RWTH Aachen University; by the Else Kröner-Fresenius-Stiftung (2014_A193; to W.W.); and by the Deutsche Forschungsgemeinschaft (DFG; WA 1706/8-1, WA1706/11-1, and 363055819/GRK2415; to W.W.).

Received: February 27, 2019
Revised: December 17, 2019
Accepted: December 23, 2019
Published: January 23, 2020

REFERENCES

Bittles, A.H., and Harper, N. (1984). Increased glycolysis in ageing cultured human diploid fibroblasts. Biosci. Rep. *4*, 751–756.

Campisi, J., and d'Adda di Fagagna, F. (2007). Cellular senescence: when bad things happen to good cells. Nat. Rev. Mol. Cell Biol. *8*, 729–740.

Chong, J., Soufan, O., Li, C., Caraus, I., Li, S., Bourque, G., Wishart, D.S., and Xia, J. (2018). MetaboAnalyst 4.0: towards more transparent and integrative metabolomics analysis. Nucleic Acids Res. *46*, W486–W494.

Coppé, J.-P., Patil, C.K., Rodier, F., Sun, Y., Muñoz, D.P., Goldstein, J., Nelson, P.S., Desprez, P.-Y., and Campisi, J. (2008). Senescence-associated secretory phenotypes reveal cell-nonautonomous functions of oncogenic RAS and the p53 tumor suppressor. PLoS Biol. *6*, e301.

Diederichs, S., and Tuan, R.S. (2014). Functional comparison of human-induced pluripotent stem cell-derived mesenchymal cells and bone marrow-derived mesenchymal stromal cells from the same donor. Stem Cells Dev. *23*, 1594–1610.

Dominici, M., Le Blanc, K., Mueller, I., Slaper-Cortenbach, I., Marini, F., Krause, D., Deans, R., Keating, A., Prockop, D., and Horwitz, E. (2006). Minimal criteria for defining multipotent mesenchymal stromal cells. The International Society for Cellular Therapy position statement. Cytotherapy *8*, 315–317.

Franzen, J., Zirkel, A., Blake, J., Rath, B., Benes, V., Papantonis, A., and Wagner, W. (2017). Senescence-associated DNA methylation is stochastically acquired in subpopulations of mesenchymal stem cells. Aging Cell *16*, 183–191.

Frobel, J., Hemeda, H., Lenz, M., Abagnale, G., Joussen, S., Denecke, B., Sarić, T., Zenke, M., and Wagner, W. (2014). Epigenetic rejuvenation of mesenchymal stromal cells derived from induced pluripotent stem cells. Stem Cell Reports *3*, 414–422.

Ho, A.D., Wagner, W., and Franke, W. (2008). Heterogeneity of mesenchymal stromal cell preparations. Cytotherapy *10*, 320–330.

James, E.L., Michalek, R.D., Pitiyage, G.N., De Castro, A.M., Vignola, K.S., Jones, J., Mohney, R.P., Karoly, E.D., Prime, S.S., and Parkinson, E.K. (2015). Senescent human fibroblasts show increased glycolysis and redox homeostasis with extracellular metabolomes that overlap with those of irreparable DNA damage, aging, and disease. J. Proteome Res. *14*, 1854–1871.

James, E.L., Lane, J.A.E., Michalek, R.D., Karoly, E.D., and Parkinson, E.K. (2016). Replicatively senescent human fibroblasts reveal a distinct intracellular metabolic profile with alterations in NAD+ and nicotinamide metabolism. Sci. Rep. *6*, 38489.

Kang, R., Zhou, Y., Tan, S., Zhou, G., Aagaard, L., Xie, L., Bünger, C., Bolund, L., and Luo, Y. (2015). Mesenchymal stem cells derived from human induced pluripotent stem cells retain adequate osteogenicity and chondrogenicity but less adipogenicity. Stem Cell Res. Ther. *6*, 144.

Koch, C.M., Joussen, S., Schellenberg, A., Lin, Q., Zenke, M., and Wagner, W. (2012). Monitoring of cellular senescence by DNA-methylation at specific CpG sites. Aging Cell *11*, 366–369.

Koch, C.M., Reck, K., Shao, K., Lin, Q., Joussen, S., Ziegler, P., Walenda, G., Drescher, W., Opalka, B., May, T., et al. (2013). Pluripotent stem cells escape from senescence-associated DNA methylation changes. Genome Res. *23*, 248–259.

Lapasset, L., Milhavet, O., Prieur, A., Besnard, E., Babled, A., Aït-Hamou, N., Leschik, J., Pellestor, F., Ramirez, J.-M., De Vos, J., et al. (2011). Rejuvenating senescent and centenarian human cells by reprogramming through the pluripotent state. Genes Dev. *25*, 2248–2253.

Lee, S.J., Yi, T., Ahn, S.H., Lim, D.K., Hong, J.Y., Cho, Y.K., Lim, J., Song, S.U., and Kwon, S.W. (2014). Senescing human bone-marrow-derived clonal mesenchymal stem cells have altered lysophospholipid composition and functionality. J. Proteome Res. *13*, 1438–1449.

Lenz, M., Goetzke, R., Schenk, A., Schubert, C., Veeck, J., Hemeda, H., Koschmieder, S., Zenke, M., Schuppert, A., and Wagner, W. (2015). Epigenetic biomarker to support classification into pluripotent and non-pluripotent cells. Sci. Rep. *5*, 8973.

Li, Y., Wu, Q., Wang, Y., Li, L., Bu, H., and Bao, J. (2017). Senescence of mesenchymal stem cells (Review). Int. J. Mol. Med. *39*, 775–782.

Long, T., Hicks, M., Yu, H.-C., Biggs, W.H., Kirkness, E.F., Menni, C., Zierer, J., Small, K.S., Mangino, M., Messier, H., et al. (2017). Whole-genome sequencing identifies common-to-rare variants associated with human blood metabolites. Nat. Genet. *49*, 568–578.

Rasko, J.E.J., Patel, A., Griffin, J.E., Gilleece, M.H., Radia, R., Yeung, D.T., Slukvin, I., Kelly, K., and Bloor, A.J. (2019). Results of the first completed clinical trial of an iPSC-derived product: CYP-001 in steroid-resistant acute GvHD. Biol. Blood Marrow Transplant. *25*, S255–S256.

Sabapathy, V., and Kumar, S. (2016). hiPSC-derived iMSCs: NextGen MSCs as an advanced therapeutically active cell resource for regenerative medicine. J. Cell. Mol. Med. *20*, 1571–1588.

Schellenberg, A., Mauen, S., Koch, C.M., Jans, R., de Waele, P., and Wagner, W. (2014). Proof of principle: quality control of therapeutic cell preparations using senescence-associated DNA-methylation changes. BMC Res. Notes *7*, 254.

Tufi, R., Gandhi, S., de Castro, I.P., Lehmann, S., Angelova, P.R., Dinsdale, D., Deas, E., Plun-Favreau, H., Nicotera, P., Abramov, A.Y., et al. (2014). Enhancing nucleotide metabolism protects against mitochondrial dysfunction and neurodegeneration in a PINK1 model of Parkinson's disease. Nat. Cell Biol. *16*, 157–166.

Wagner, W., and Ho, A.D. (2007). Mesenchymal stem cell preparations - comparing apples and oranges. Stem Cell Rev. *3*, 239–248.

Wagner, W., Horn, P., Castoldi, M., Diehlmann, A., Bork, S., Saffrich, R., Benes, V., Blake, J., Pfister, S., Eckstein, V., et al. (2008). Replicative senescence of mesenchymal stem cells: a continuous and organized process. PLoS One *3*, e2213.

Watanabe, S., Kawamoto, S., Ohtani, N., and Hara, E. (2017). Impact of senescence-associated secretory phenotype and its potential as a therapeutic target for senescence-associated diseases. Cancer Sci. *108*, 563.

Wiley, C.D., and Campisi, J. (2016). From ancient pathways to aging cells - connecting metabolism and cellular senescence. Cell Metab. *23*, 1013–1021.

Willmann, C.A., Hemeda, H., Pieper, L.A., Lenz, M., Qin, J., Joussen, S., Sontag, S., Wanek, P., Denecke, B., Schüler, H.M., et al. (2013). To clone or not to clone? Induced pluripotent stem cells can be generated in bulk culture. PLoS One *8*, e65324.

Xia, J., Sinelnikov, I.V., Han, B., and Wishart, D.S. (2015). MetaboAnalyst 3.0–making metabolomics more meaningful. Nucleic Acids Res. *43*, W251–W257.

Yin, S., Dragusha, S., Ejupi, V., Shibata, T., Kabashima, T., and Kai, M. (2015). Sensitive and selective determination of orotic acid in biological specimens using a novel fluorogenic reaction. J. Fluoresc. *25*, 1005–1011.

Zhang, H., Menzies, K.J., and Auwerx, J. (2018). The role of mitochondria in stem cell fate and aging. Development *145*, dev143420.

Zwerschke, W., Mazurek, S., Stöckl, P., Hütter, E., Eigenbrodt, E., and Jansen-Dürr, P. (2003). Metabolic analysis of senescent human fibroblasts reveals a role for AMP in cellular senescence. Biochem. J. *376*, 403–411.

Stem Cell Reports, Volume *14*

Supplemental Information

Senescence-Associated Metabolomic Phenotype in Primary and iPSC-Derived Mesenchymal Stromal Cells

Eduardo Fernandez-Rebollo, Julia Franzen, Roman Goetzke, Jonathan Hollmann, Alina Ostrowska, Matteo Oliverio, Torsten Sieben, Björn Rath, Jan-Wilhelm Kornfeld, and Wolfgang Wagner

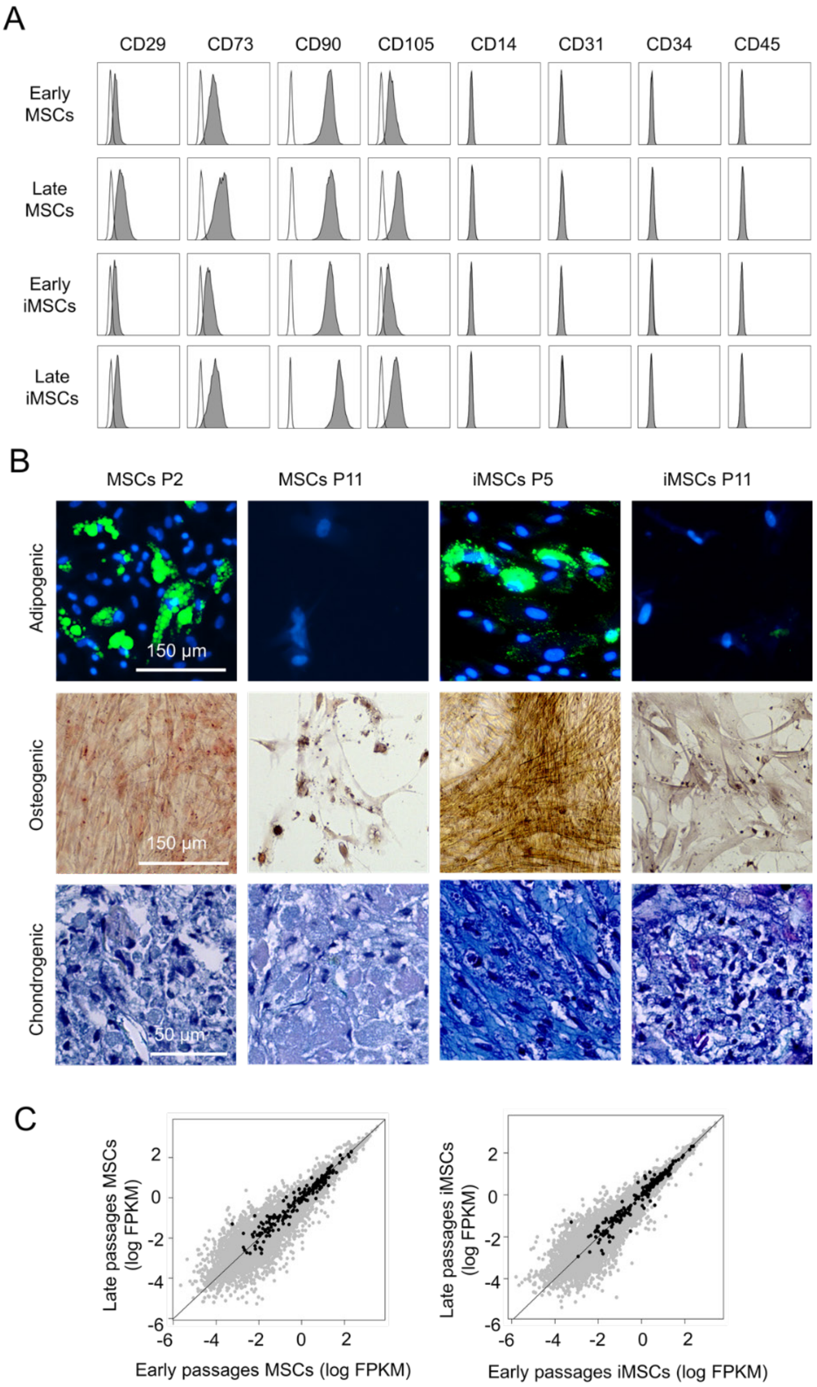

Figure S1: Immunophenotype and Differentiation Potential of MSCs and iMSCs, Related to Figure 1. (A) Histograms depict exemplarily the immunophenotype of MSCs and iMSCs in early and late passages. **(B)** MSCs and iMSCs in early and late passages were differentiated towards adipogenic, osteogenic and chondrogenic lineages and analyzed as indicated in the text. **(C)** Expression of the genes involved in the senescence associated secretory phenotype (SASP). To that end we curated a list of genes - combining the list of genes from http://www.saspatlas.com/, https://reactome.org and https://www.labome.com/, which have been associated to SASP. Scatter plot of early versus late passages in MSCs (left) and iMSCs (right) indicated that SASP associated genes (black dots) are not generally upregulated as compared to other genes (grey). There was overall little up-regulation of genes for SASP proteins, which might be attributed to difference between gene expression and protein level, and to heterogeneity amongst cell types and time points (Hernandez-Segura et al., 2017).

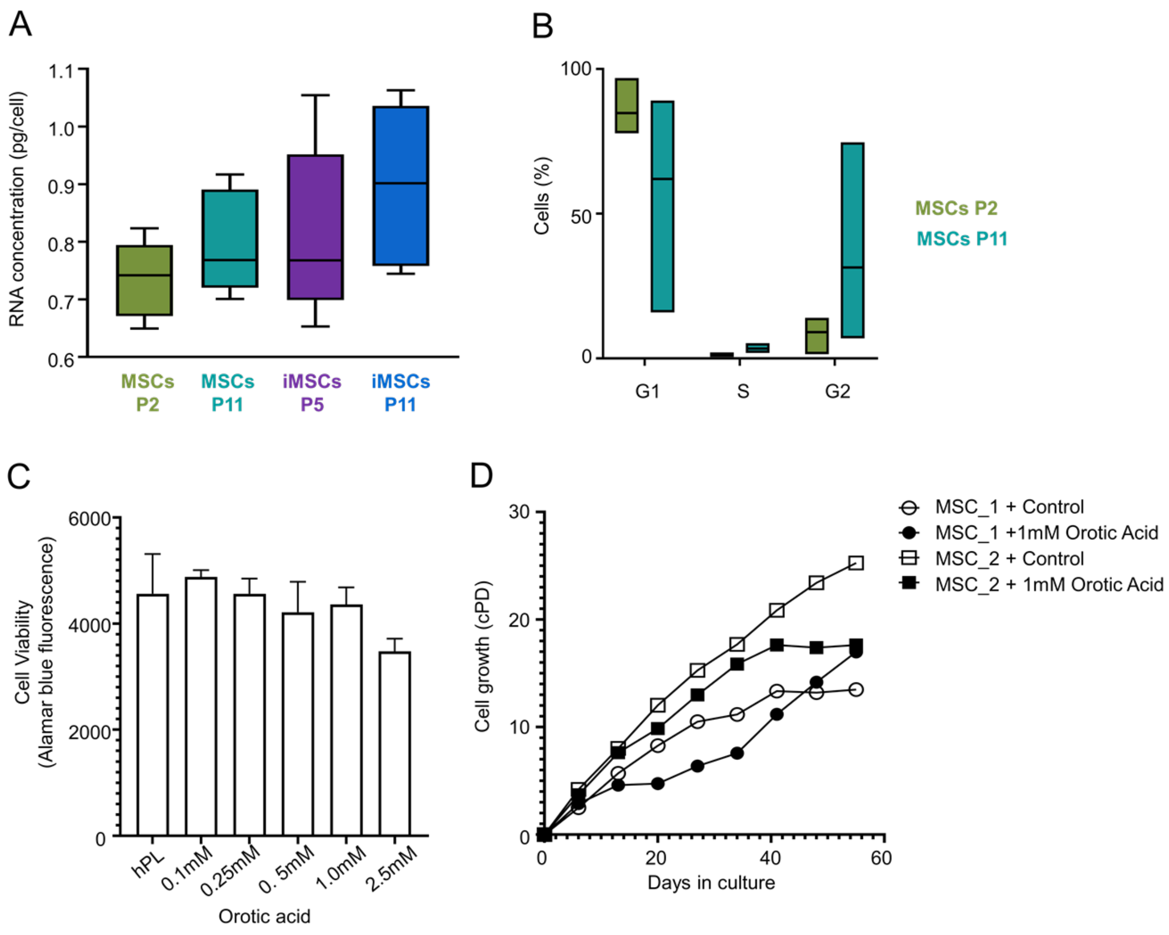

Figure S2: Cell Cycle Arrest and Culture with Orotic Acid, Related to Figure 3.
It has been suggested that nucleotide synthesis inhibition plays a causative role in the establishment of replicative senescence in human mammary epithelial cells (Delfarah et al., 2019). Therefore, we analyzed the RNA concentration per cell, the cell cycle, and the impact of orotic acid, which is needed for pyrimidine synthesis. **(A)** Box plot representation for normalized RNA content (pg/cell) for MSCs and iMSCs in early and late passages (n = 5). The higher RNA concentration might be related to the increasing cell size of senescent cells. **(B)** Cell cycle analysis was performed by flow cytometry with Propidium Iodide intercalation. The proportion of cells in G2 increase in MSCs of later passage (n = 3), which is in line with previous reports (Gire and Dulić, 2015; Mao et al., 2012). **(C)** For supplementation experiments with orotic acid, we have initially tested the concentration that did not impair viability after three days (Alamar blue assay; n = 3). **(D)** 1 mM orotic acid was subsequently substituted to MSC culture media and there was no consistent impact on long-term growth curves of two MSC preparations (cPD = cumulative population doublings).

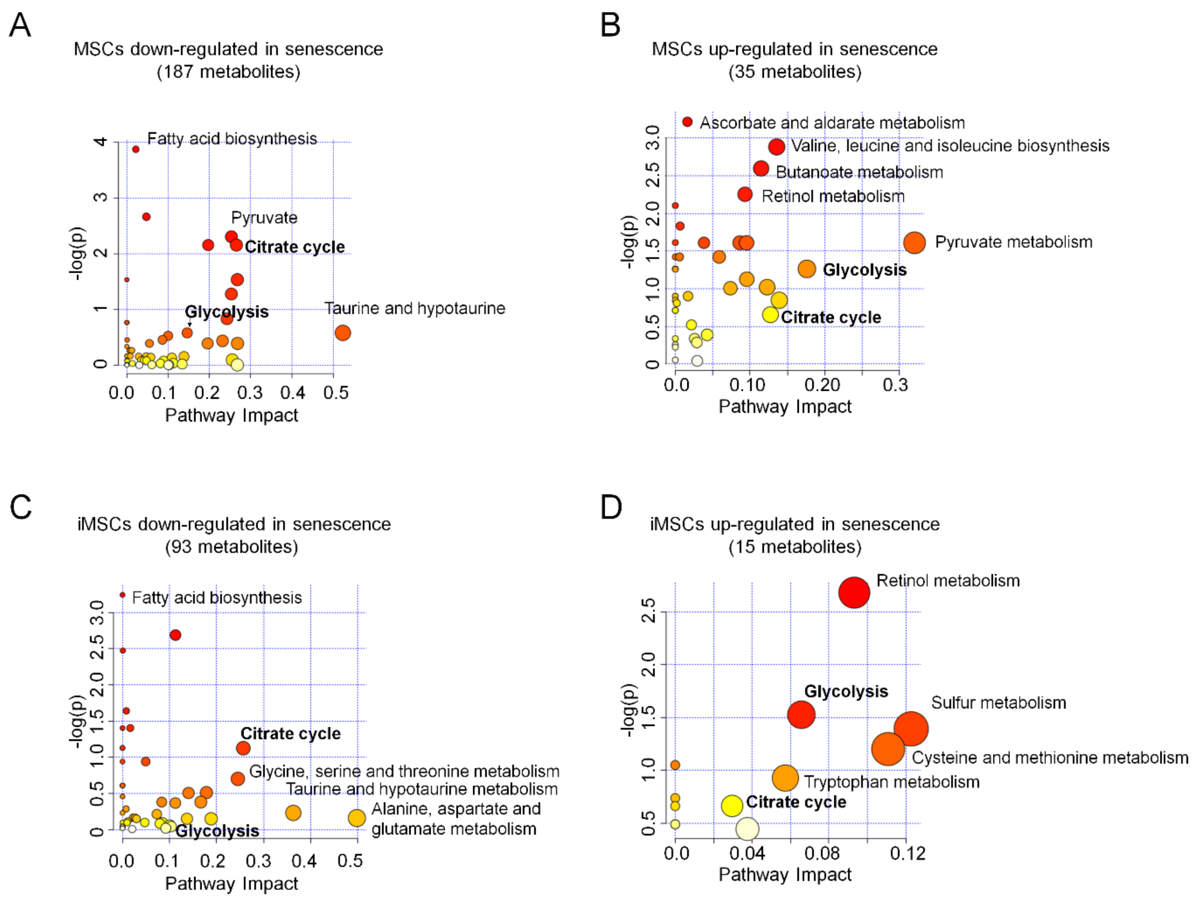

Figure S3: Metabolic Pathway Analysis in either MSC or iMSC, Related to Figure 4.
The metabolic pathway analysis (using MetaboAnalyst 4.0) was performed for metabolites that were either down- or up-regulated during replicative senescence of either MSCs **(A, B)** or iMSCs **(C, D)**. Scores for enrichment (vertical axis) and topology analyses (pathway impact, horizontal axis) are depicted (color code depicts overall significance, and the size of the circles reflects centrality of the involved metabolites).

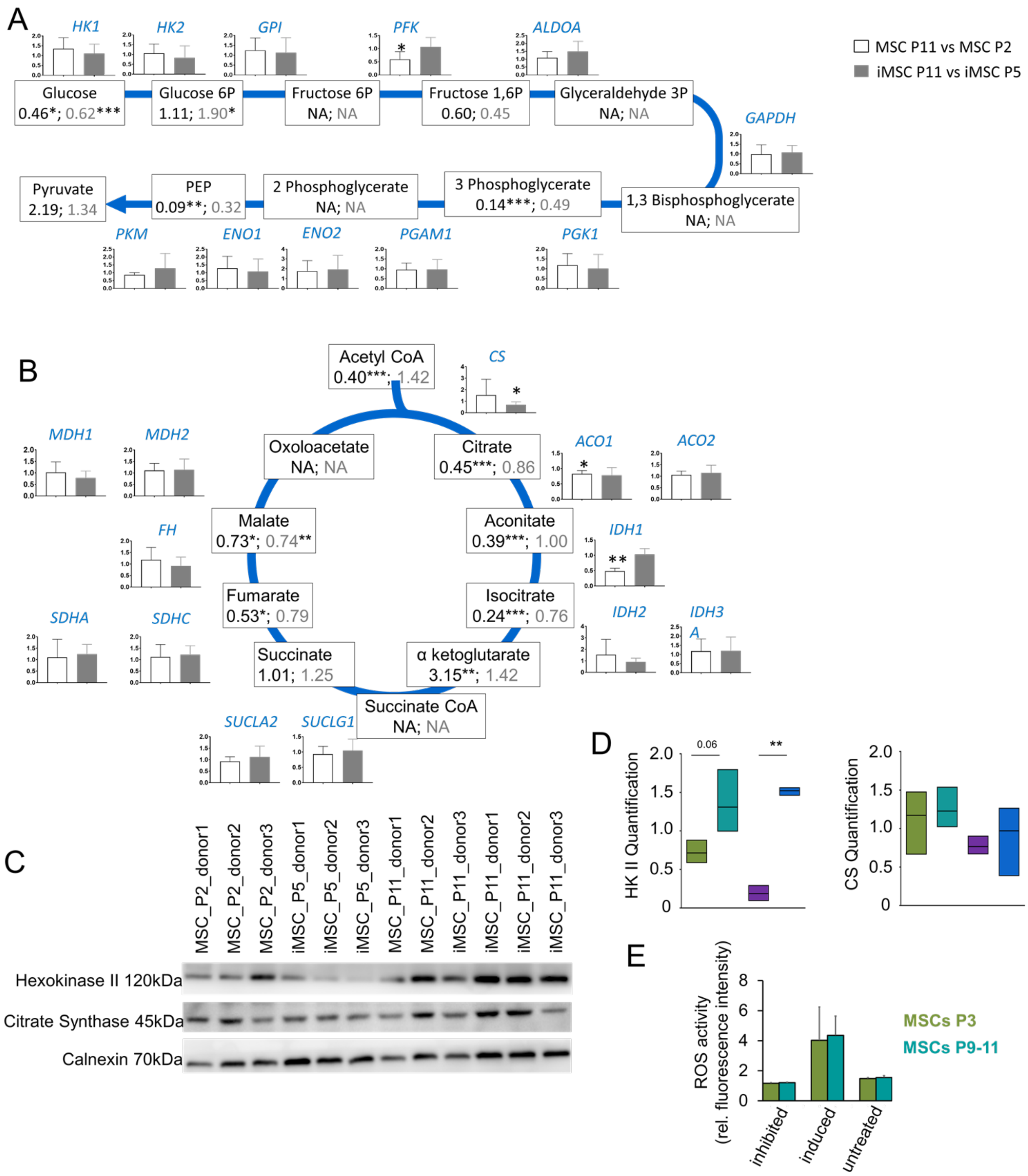

Figure S4: Changes in Glycolysis and Tricarboxylic Acid Cycle, Related to Figure 4.
Senescence-associated gene expression changes of enzymes involved in glycolysis **(A)** and TCA cycle **(B)** are represented by bars (MSCs P11 versus MSCs P2 in white; and iMSCs P11 versus iMSCs P5 in grey). In addition, the corresponding fold-changes of metabolites are indicated in the boxes (for MSCs and iMSCs, respectively). The discrepancies between gene expression and metabolites might be attributed to additional impact on mRNA splicing, translation, posttranslational modification, protein activation and localization. Abbreviations: HK, hexokinase; GPI, glucose-6-phosphate isomerase; PFK, phosphofructokinase; ALDOA, aldolase A; GAPDH, glyceraldehyde 3-phosphate dehydrogenase; PGK1, phosphoglycerate kinase; PGAM1, phosphoglycerate mutase 1; ENO enolase; PKM, pyruvate kinase; CS, citrate synthase; ACO, aconitase; FH, fumarate hydratase; IDH, isocitrate dehydrogenase; MDH,

malate dehydrogenase; SUCL, succinate/malate CoA ligase; SDH, succinate dehydrogenase; NA, not available. **(C)** Western Blots depict exemplarily for two key proteins in Glycolysis (Hexokinase II) and TCA (Citrate Synthase) as well as the housekeeping (Calnexin). **(D)** Relative quantification of Western Blots (n = 3; two technical replica each) reveals increased Hexokinase II (HK II) levels in late passages for MSCs and iMSCs, while no changes for Citrate Synthase (CS) levels. **(E)** Cellular reactive oxygen levels (ROS) demonstrated a slight increase of ROS levels at later passages – despite the metabolic shift – which is in line with previous publications (Geißler et al., 2012; Jeong and Cho, 2015). Fluorescence intensity (488/520 nm) of ROS detection reagent was measured for three different MSC donors at early and late passage (six technical replica for each donor). ROS was inhibited with N-acetyl-L-cysteine and induced using Pyocyanin following the manufacturer´s instructions. The untreated cells did not reveal a clear increase in ROS levels at later passages. In analogy we analyzed superoxide levels, but we did not observe changes in superoxide levels in late passages (data not shown). All results were normalized to unstained cells.

Supplemental Tables

Supplemental Table S1: Differentially Expressed Transcripts upon Senescence of MSCs and iMSCs, Related to Figure 2.
This table is provided as separate EXCEL File

Supplemental Table S2: Identified Metabolites, Related to Figure 3.
This table is provided as separate EXCEL File

Supplemental Methods

Flow Cytometry

Immunophenotypic surface marker analysis was performed on a FACS Canto II (BD, Heidelberg, Germany) upon staining with CD14 allophycocyanin (APC, clone M5E2; BD), CD29 phycoerythrin (PE, clone MAR4; BD), CD31 PE (clone WM59; BD), CD34 APC (clone 8G12; BD), CD45 APC (clone HI30; BD), CD73 PE (clone AD2; BD), CD90 APC (clone 5E10; BD), CD105 fluorescein isothiocyanate (FITC, clone MEM-226; ImmunoTools, Friesoythe, Germany). Autofluorescence was used as negative control.

Analysis of Proliferation

At every passage cells were harvested by trypsinization, counted in a Neubauer cell chamber and seeded in defined numbers. Cumulative population doublings (cPDs) were calculated as described before (Cholewa et al., 2011).

In vitro differentiation of MSCs and iMSCs

Differentiation of MSCs and iMSCs toward adipogenic, osteogenic, and chrondrogenic lineage was induced as previously described (Frobel et al., 2014). After three weeks, osteogenic differentiation of MSCs and iMSCs was analyzed by staining of calcium precipitates with Alizarin Red S and quantified on a Tecan Infinite 200 plate reader (Gregory et al., 2004). Fat droplets in adipogenic differentiation were stained with BODIPY counterstained with DAPI. Glycosaminoglycan deposition in chondrogenic differentiation was analyzed by Alcian Blue and PAS staining.

Transcriptomics

RNA was isolated from the samples using the NucleoSpin RNA extraction kit (Macherey-Nagel, Düren, Germany). Library preparation was performed with the Illumina TruSeq Stranded Total RNA Sample Preparation Kit, with a total input amount of 1 µg, following the standard protocol with ribosomal depletion (Illumina Ribo-Zero Gold rRNA removal kit) and the NextSeq 500/550 High Output Kit v2 (carried out by the Core Genomics-facility IZKF, RWTH Aachen University). The resulting pair-end reads were quality-checked with FastQC (http://www.bioinformatics.babraham.ac.uk/projects/fastqc/), and low-quality reads were removed using TrimGalore. Reads were mapped to the GRCh38 assembly of the human genome using Tophat2 (Trapnell et al., 2009), version 2.0.10, and reassembled with Cufflinks (Trapnell et al., 2012), version 2.1.1. Differential gene expression was analyzed using the DESeq2 package (Love et al., 2014), version 1.10.1. Significance of transcriptomic changes was estimated using the R limma package with an adjusted paired p-value <0.05. Gene Ontology analysis was performed with GoMiner™.

Immunoblot Analysis

For protein isolation, 100 µl of RIPA lysis buffer (50 mM Tris-HCL pH 7.5, 150 mM NaCl 1 mM EDTA, 0.1 % sodium deoxycholate, 1 % NP-40, 1x protease inhibitor (Sigma) and 1 x phosphatase inhibitor (Roche)) was added to 1 million cells. Protein lysates were transferred to 1.5 ml Eppendorf tubes and snap-frozen in liquid nitrogen and thawed on ice for three repeated cycles. After 10 min of centrifugation at 12,000 g and 4° C the supernatant of all samples was collected into fresh tubes and and protein concentration was determined using the Bradford colorimetric protein method (Thermo Fisher Scientific). Protein samples were stored at -80 °C. Samples for immunoblotting were separated by SDS-PAGE after being prepared with 4x Laemmli Sample Buffer containing 10 % β-mercaptoethanol and heated to 95° C for 5 min. Subsequently, proteins were transferred to nitrocellulose membrane and blocked with Western Blocking Reagent (Roche). Membranes were incubated with primary antibodies at 4° C overnight. After incubation with secondary antibodies, blots were developed with Pierce ECL Western Blotting substrate (Thermo Fisher Scientific). All densitometry measurements were performed with ImageJ. Band intensity was normalized to calnexin (CLNX), which served as internal loading control, blotted on the same gel and membrane. Citrate synthase antibody was purchased from Cell Signaling Technology (catalogue no. 14309), while hexokinase II antibody was purchased from Abcam (catalogue no. (ab131196). Primary antibodies were used at a 1:1000 dilution ratio in Tween TBS. Anti-CLNX antibody was used at a 1:5,000 dilution and purchased from Calbiochem (catalogue no. 208-880).

Quantification of orotic acid

A confluent well of a six-well plate was used to isolate the metabolites with methanol (Ser et al., 2015), while another well was used to determine the total protein concentration to normalize the results . Briefly, 50 µL of the sample was mixed with 50 µL of 4.0 mM of 4-TFMBAO, 50 µL of 8.0 mM $K_3[Fe(CN)_6]$ and 50 µL of 80 mM K_2CO_3. The mixture (200 µL) was then heated at 80 °C for 4 min, followed by cooling in an ice bath for approx. 2 min to stop the reaction. The relative fluorescence intensity produced by the reaction with 4-TFMBAO was measured with TECAN 200 plate reader (Tecan Group Ltd., Switzerland) at maximum excitation and emission wavelengths of 340 and 460 nm, respectively.

Metabolic flux analysis

The XFe96 Flux analyzer and Prep Station (Seahorse Bioscience XFe96 Instrument, Agilent, CA, USA) were used according to the manufacturer's instructions (Ferrick et al., 2008). Briefly, cells were seeded in XFe96 cell culture plates at 25,000 cells per well and cultured overnight. The XFe96 sensor cartridges were hydrated overnight with 200 µL of Seahorse Bioscience XFe96 Calibrant at pH 7.4 and stored at 37 °C without CO_2. One hour before the measurement, cells were washed and culture media were replaced with no-glucose media. Basal measurements of ECAR and OCR, as well as measurements after addition of glucose (final concentration 10 mM; Sigma Aldrich, St. Louis, MO, USA), oligomycin (final

concentration 5 µM; Sigma Aldrich) and 2-deoxy-d-glucose (2DG, final concentration 100 mM; Seahorse Bioscience), were performed as described in the XF Glycolysis Stress Test Kit User Manual (Seahorse Bioscience).

Cellular ROS/Superoxide Detection Assay Kit

Oxidative stress production was investigated through Cellular ROS/Superoxide detection assay kit (ab139476, Abcam, Cambridge, UK) following the manufacturer's protocol. Briefly, MSCs and iMSCs cells were seeded (1 x 10^4 cells/well) into transparent 96-well plates and maintained to attach at 37 °C in 5% CO2. After 24 h cells were pre-incubated with superoxide detection reagent (orange) or ROS detection reagent (green) for 1 h. After that, the fluorescence intensity was measured with a Tecan Infinite 200 plate reader (Tecan Group Ltd., Männedorf, Switzerland), with an excitation and emission wavelengths of 550/610 nm and for Superoxide detection and 488/520 nm to detect ROS.

Supplemental References

Cholewa, D., Stiehl, T., Schellenberg, A., Bokermann, G., Joussen, S., Koch, C., Walenda, T., Pallua, N., Marciniak-Czochra, A., Suschek, C. V, et al. (2011). Expansion of adipose mesenchymal stromal cells is affected by human platelet lysate and plating density. Cell Transplant. *20*, 1409–1422.

Delfarah, A., Parrish, S., Junge, J.A., Yang, J., Seo, F., Li, S., Mac, J., Wang, P., Fraser, S.E., and Graham, N.A. (2019). Inhibition of nucleotide synthesis promotes replicative senescence of human mammary epithelial cells. J. Biol. Chem. *294*, 10564–10578.

Ferrick, D.A., Neilson, A., and Beeson, C. (2008). Advances in measuring cellular bioenergetics using extracellular flux. Drug Discov. Today *13*, 268–274.

Frobel, J., Hemeda, H., Lenz, M., Abagnale, G., Joussen, S., Denecke, B., Sarić, T., Zenke, M., and Wagner, W. (2014). Epigenetic rejuvenation of mesenchymal stromal cells derived from induced pluripotent stem cells. Stem Cell Reports *3*, 414–422.

Geißler, S., Textor, M., Kühnisch, J., Könnig, D., Klein, O., Ode, A., Pfitzner, T., Adjaye, J., Kasper, G., and Duda, G.N. (2012). Functional Comparison of Chronological and In Vitro Aging: Differential Role of the Cytoskeleton and Mitochondria in Mesenchymal Stromal Cells. PLoS One *7*, e52700.

Gire, V., and Dulić, V. (2015). Senescence from G2 arrest, revisited. Cell Cycle *14*, 297–304.

Gregory, C.A., Gunn, W.G., Peister, A., and Prockop, D.J. (2004). An Alizarin red-based assay of mineralization by adherent cells in culture: comparison with cetylpyridinium chloride extraction. Anal. Biochem. *329*, 77–84.

Hernandez-Segura, A., de Jong, T. V., Melov, S., Guryev, V., Campisi, J., and Demaria, M. (2017). Unmasking Transcriptional Heterogeneity in Senescent Cells. Curr. Biol. *27*, 2652-2660.e4.

Jeong, S.-G., and Cho, G.-W. (2015). Endogenous ROS levels are increased in replicative senescence in human bone marrow mesenchymal stromal cells. Biochem. Biophys. Res. Commun. *460*, 971–976.

Love, M.I., Huber, W., and Anders, S. (2014). Moderated estimation of fold change and dispersion for RNA-seq data with DESeq2. Genome Biol. *15*, 550.

Mao, Z., Ke, Z., Gorbunova, V., and Seluanov, A. (2012). Replicatively senescent cells are arrested in G1 and G2 phases. Aging (Albany. NY). *4*, 431–435.

Ser, Z., Liu, X., Tang, N.N., and Locasale, J.W. (2015). Extraction parameters for metabolomics from cultured cells. Anal. Biochem. *475*, 22–28.

Trapnell, C., Pachter, L., and Salzberg, S.L. (2009). TopHat: discovering splice junctions with RNA-Seq. Bioinformatics *25*, 1105–1111.

Trapnell, C., Roberts, A., Goff, L., Pertea, G., Kim, D., Kelley, D.R., Pimentel, H., Salzberg, S.L., Rinn, J.L., and Pachter, L. (2012). Differential gene and transcript expression analysis of RNA-seq experiments with TopHat and Cufflinks. Nat. Protoc. *7*, 562–578.
